# Hyaluronic Acid-Coated SPIONs with Attached Folic Acid as Potential T2 MRI Contrasts for Anticancer Therapies

**DOI:** 10.1021/acsami.4c20101

**Published:** 2025-01-29

**Authors:** Martyna Kasprzyk, Gabriela Opiła, Alicja Hinz, Sylwia Stankiewicz, Monika Bzowska, Karol Wolski, Joanna Dulińska-Litewka, Janusz Przewoźnik, Czesław Kapusta, Anna Karewicz

**Affiliations:** † Faculty of Chemistry, Jagiellonian University, Gronostajowa 2, 30-387 Kraków, Poland; ‡ Doctoral School of Exact and Natural Sciences, Jagiellonian University, Prof. S. Łojasiewicza 11, 30-348 Kraków, Poland; § Faculty of Physics and Applied Computer Science, AGH University of Kraków, Al. A. Mickiewicza 30, 30-059 Kraków, Poland; ∥ Department of Cell Biochemistry, Faculty of Biochemistry, Biophysics and Biotechnology, Jagiellonian University, Gronostajowa 7, 30-387 Kraków, Poland; ⊥ Chair of Medical Biochemistry, Jagiellonian University Medical College, Kopernika 7, 31-034 Kraków, Poland

**Keywords:** magnetic nanoparticles, hyaluronic acid, MRI contrast, folic acid, glioblastoma

## Abstract

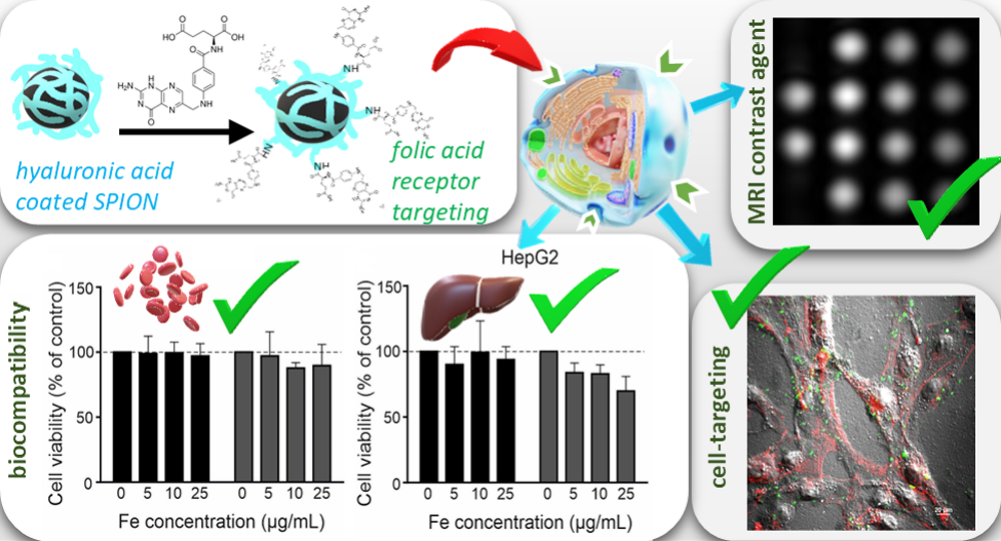

Superparamagnetic iron oxide nanoparticles (SPIONs) are known to be good MRI contrasts, but they have a high tendency to aggregate and their biocompatibility is limited. Hyaluronic acid is highly biocompatible, can provide SPION with colloidal stability, and interacts specifically with tumor cells through the CD44 receptor; therefore, it was used as a stabilizing layer. We successfully obtained SPION coated with hyaluronic acid and further functionalized it with folic acid to construct a dual-targeted system. The physicochemical properties of the nanoparticles were investigated using DLS/ELS, AFM, XRD, and ATR-FTIR. Their magnetic characterization was performed by magnetometry, Mössbauer spectroscopy, ^1^H NMR T1 and T2 measurements, and MRI. The nanoparticles’ biocompatibility was verified on blood and hepatocytes, and their cytotoxicity was tested on glioma and adenocarcinoma cells using the MTT assay. The nanoparticles were spherical, colloidally stable, and had low dispersity. Their cores were formed by 7 nm crystallites of magnetite in its oxidized form, maghemite. Our SPIONs were superparamagnetic and could potentially serve as effective T2 contrasts for MRI. The performance of SPIONs modified with folic acid was superior to that observed for commercial contrasts. Our nanoparticles were also hemocompatible and were efficiently taken up by glioblastoma cancer cells. Folic acid-modified SPIONs could also reduce viability of tumor cells in a dose-dependent manner. Thus, the proposed system has potential application as both a diagnostic tool and a therapeutic agent for targeted anticancer therapies.

## 1 Introduction

According to WHO statistics, cancer is the second leading cause of death, with some 20 million new cases and 9.7 million cancer-related deaths worldwide. Projections are even more alarming, with the WHO predicting that by 2050, the number of new cancer cases will exceed 35 million. While the concerted efforts of the scientific community are focused on developing new, more effective anticancer therapies, there is also an intense search for precise and safe diagnostic tools for early detection, accurate diagnosis, and monitoring of cancer treatment.^1,2^

Magnetic resonance imaging (MRI) is a noninvasive imaging technique that produces three-dimensional, detailed anatomical images with high spatial resolution and contrast. It is routinely used by physicians to detect and evaluate solid tumors, including but not limited to brain,^3^ lung,^4^ breast, and pancreatic cancer.^5^ MRI contrast agents significantly increase the image contrast between normal and diseased tissues by altering the longitudinal (T1) and transverse (T2) relaxation rates of the surrounding water proton spins. They are classified as T1 positive contrast agents or T2 negative contrast agents based on their magnetic properties and relaxation mechanisms. While T1 contrast agents are usually based on gadolinium complexes,^6^ superparamagnetic iron oxide nanoparticles are usually proposed as T2 contrasts.^7−9^

SPIONs are iron oxide nanocrystals, usually composed of magnetite or its oxidized form, maghemite, with a size of less than 20 nm, which are characterized by unique magnetic properties. Unlike bulk magnetite, SPIONs at room temperature usually do not exhibit a hysteresis loop as a function of the external magnetic field as each small crystal represents a single magnetic domain, which undergoes thermal fluctuations. Due to their high tendency to aggregate in aqueous environments, SPIONs must be coated, usually with surfactants or polymers, to ensure their colloidal stability.^10^ The introduced coating can significantly affect not only the physicochemical properties of SPIONs but also their magnetic behavior^11^ and biological interactions.^12^ This may also allow for surface functionalization to enhance their biocompatibility or target SPIONs to specific tissues or cells.

Hyaluronic acid is a naturally occurring polysaccharide distributed in connective, epithelial, and neural tissues. As a component of the extracellular matrix, it affects cell proliferation and migration. Hyaluronic acid is highly biocompatible and modulates important functions of cancer cells through interactions with the CD44 receptor and RHAMM (receptor for hyaluronic acid-mediated motility),^13^ which are overexpressed in many types of cancer: head and neck,^14^ gliomas,^13,15^ breast, colorectal, pancreatic, ovarian, thyroid, and endometrial cancers.^14^ The CD44 receptor is also expressed in normal tissue cells but at low levels.^16^ Spadea et al.^14^ also confirmed that HA has a higher affinity for CD44 receptors expressed in cancer cells than for those in normal cells. These properties make HA an excellent coating for SPION-based MRI contrasts. So far, there are only a few reports of HA-stabilized SPIONs. In some of them, the nanoparticles were first modified with a positively charged coating, such as dopamine^17^ or PEI,^18^ and HA was attached as a second layer. Another approach was presented by Lachowicz et al.,^19^ where SPIONs were first stabilized with a layer of a cationic chitosan derivative, and then coated with a conjugate of HA and curcumin (HA-Cur). The introduction of the HA-Cur conjugate significantly increased the T2 of the obtained SPIONs. Zhang et al.^20^ and Soleymani et al.^21^ used direct HA coating, but their approaches required extended time and high temperatures.

Here, we propose a new nanoparticle system based on HA-stabilized SPIONs obtained by direct coprecipitation of iron(II) and iron(III) oxides in the presence of HA under relatively mild conditions, followed by surface modification with folic acid (FA). It is known that FA is a ligand for the proton-coupled folic acid transporter (PCFT), the expression of which is 500-fold higher in cancer cells than in healthy cells, especially in gliomas.^22,23^ Due to the presence of the HA coating and the proposed synthesis method, our nanoparticles are highly biocompatible, colloidally stable, and exhibit unique magnetic properties that allow them to act as T2 MRI contrasts. At the same time, the presence of both HA coating and FA ligand greatly enhances their ability to target various tumor cells via CD44, RHAMM, or folate receptors, increasing their potential as MRI contrasts for cancer diagnosis and treatment monitoring.

## 2 Experimental Section

### 2.1 Materials

Hyaluronic acid sodium salt (Na-HA; Mw = (30–50) kDa, purity ≥91.0%) was purchased from Pol-Aura (Zabrze, Poland). Folic acid (FA; purity 97%), cystamine dihydrochloride (CYS; purity 96.0%), dimethyl sulfoxide (DMSO; anhydrous, purity ≥99.0%), fluorescein isothiocyanate (FITC; BioReagent; ≥90%), iron(II) chloride tetrahydrate (puriss. p.a., ≥98.5%), iron(III) chloride hexahydrate (puriss. p.a., ≥98.5%), *N*-(3-(dimethylamino)propyl)-*N*′-ethylcarbodiimide hydrochloride (EDC; purity ≥98%), *N*-hydroxysuccinimide (NHS; purity ≥98%), phosphate-buffer saline (PBS; tablets; pH = 7.4), picrylsulfonic acid solution (TNBS; 5% (w/v) in H_2_O; BioReagent, suitable for determination of primary amines), and triethylamine (TEA; purity ≥99%) were purchased from Merck (Warsaw, Poland). Hoechst 33342 (NucBlue Live ReadyProbes) was purchased from ThermoFisher Scientific (Warsaw, Poland). Phalloidin-CF633 conjugate (Biotium) was purchased from VWR (Gdansk, Poland). Ammonia solution (p.a., 5 M) was obtained from POCH S.A. (Gliwice, Poland). All of the reagents were used as received.

### 2.2 Methods

#### 2.2.1 Preparation of HA-Coated SPIONs (SPION/HA)

HA-coated SPIONs were synthesized by coprecipitation of iron(II) and iron(III) chlorides in the presence of the hyaluronic acid sodium salt (Na-HA). Briefly, Na-HA (100 mg) was dissolved in 10 mL of 0.5 M ammonia solution to obtain the final concentration of 10 mg/mL and then sonicated (Sonic-6, Polsonic, 480 W, 1 s pulse per every 5 s) for 3 h in 50 °C. The Na-HA solution was then placed on the magnetic stirrer under a constant argon flow and heated to 60 °C for 15 min. 0.0232 g of FeCl_3_·6H_2_O and 0.0085 g of FeCl_2_·4H_2_O (Fe(III):Fe(II) molar ratio 2:1) were dissolved in 1 mL of deionized water and added dropwise to the vigorously stirred solution of Na-HA, followed by a rapid addition of 4 mL of 5 M ammonia solution. The reaction was continued for 30 min. The obtained nanoparticulate suspension was purified by dialysis (MWCO 14 kDa) against deionized water for 24 h, followed by the magnetic chromatography (as described before^24^) and finally filtered using a PES syringe filter (0.45 μm). The product was stored in 4 °C.

#### 2.2.2 Surface Modification of SPION/HA with FA

**Figure 1:**
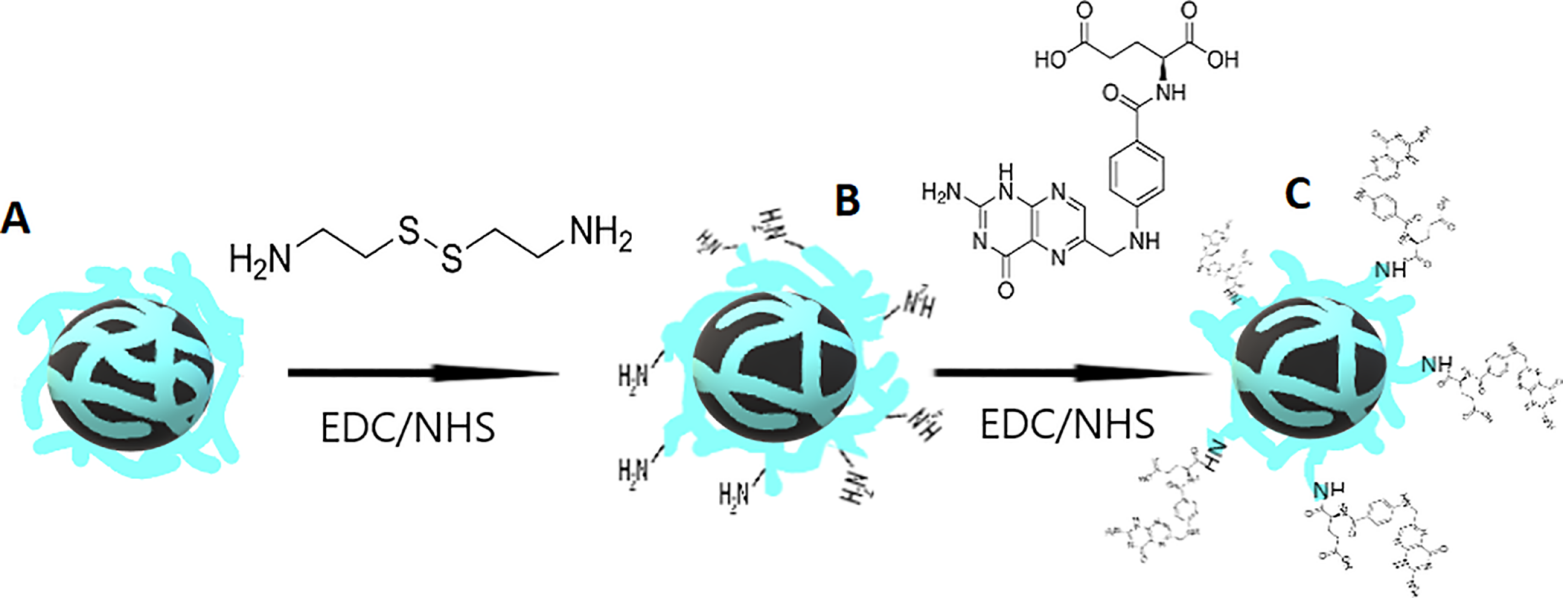
Scheme of the SPION/HA-FA synthesis, where (A) SPION/HA, (B) SPION/HA-CYS, and (C) SPION/HA-FA.

SPION/HA functionalization with FA as an additional targeting ligand was a two-step process. First, the nanoparticles were modified with CYS to introduce free amino groups into the HA shell. Then, the SPION/HA-CYS nanoparticles were further conjugated with FA.^25,26^ The schematic representation of the synthesis of SPION/HA-FA is shown in Figure 1.

The carboxyl groups of HA on the surface of SPION/HA were activated by adding 14.25 mg of EDC and 8.50 mg of NHS to 1 mL of nanoparticles. The suspension was then stirred using a laboratory shaker for 15 min at 37 °C, followed by the addition of 8.50 mg of CYS. The reaction was carried out for another 16 h. The resulting SPION/HA-CYS were purified by dialysis (MWCO 14 kDa) against deionized water for 24 h and concentrated by magnetic chromatography. The concentration of introduced primary amine groups was estimated by the TNBS method described in detail previously,^25^ using a calibration curve (*R*^2^ = 0.9999; concentrations of amine groups from 0.1 to 5.0 mM). Absorbance at 410 nm was measured by using a Cary 60 UV–Vis spectrophotometer (Agilent Technologies). The signal from SPION/HA was used as a blank. The presented results are the average of three measurements.

Then, 20 mg of FA was dissolved in 0.8 mL of anhydrous DMSO, followed by the addition of 43.43 mg of EDC, 26.07 mg of NHS and 100 μL of TEA, and stirred in the dark for 20 min. The resulting solution was added to 5 mL of SPION/HA-CYS suspension at pH = 10. pH correction was made by adding 0.2 M NaOH to the nanoparticles’ suspension. The coupling reaction was carried out for 24 h in the dark, and the resulting SPION/HA-FA were purified by dialysis (MWCO 14 kDa) against deionized water for 24 h and then concentrated by magnetic chromatography. To estimate the amount of FA attached to the surface of the nanoparticles, their suspension was centrifuged (20 min, 10,000 rpm) and resuspended in deionized water in the volume initially used for synthesis. The resulting colloid was then diluted, and its absorption at 360 nm was measured. The amount of attached FA was determined from the calibration curve (*R*^2^ = 0.9995; FA concentrations in the range of 13.8–69.0 μg/mL). The signal from SPION/HA-CYS used for the FA-coupling reaction was set as a blank. The presented results are the average of three measurements. The obtained SPION/HA-CYS and SPION/HA-FA were stored at 4 °C for future use.

#### 2.2.3 Fluorescent Labeling of the SPIONs

SPION/HA and SPION/HA-FA were fluorescently labeled with FITC to enable their visualization by confocal microscopy. To achieve this, SPION/HA were functionalized by CYS, as described above. Then, 0.65 mL of FITC solution in methanol (2 mg/mL) was added to 1 mL of colloid, and the mixture was left for 18 h at room temperature in the dark. Nanoparticles were next purified by dialysis (MWCO 14 kDa) against the mixture of water and ethanol (1:1; v/v) for 2 days.^27^ To obtain FITC-labeled SPION/HA-FA, folic acid was then attached to FITC-labeled SPION/HA-CYS following the procedure described earlier for unlabeled SPION/HA-CYS.

### 2.3 Physiochemical and Magnetic Characterization of the Obtained SPIONs

#### 2.3.1 Dynamic Light Scattering (DLS)

The hydrodynamic diameter, size distribution (PDI), and zeta potential of SPION/HA and SPION/HA-FA were measured by dynamic light scattering (DLS) method using a Malvern Zetasizer Nano-ZS apparatus (Malvern Instrument Ltd., A.P. Instruments, Warsaw, Poland). The data were recorded and analyzed using Malvern Zetasizer Nano software v.3.30.

#### 2.3.2 Stability Assessment in Physiological Conditions

Stability of the obtained SPION/HA and SPION/HA-FA nanoparticles under physiological conditions was tested using DLS measurements. The stability was tested at 37 °C in (1) PBS buffer at pH = 7.4 simulating the physiological conditions before the NPs reach the tumor site and (2) PBS buffer at pH = 5.0 simulating the conditions at the tumor site. 50 μL of the SPIONs (SPION/HA or SPION/HA-FA) were suspended in 1 mL of the PBS buffer solution and incubated at 37 °C with a constant, slight shaking (10 rpm). After the predetermined time intervals, the DLS measurement was performed to determine the average hydrodynamic diameter of the particles. The results presented here are presented as the mean value of the three independent measurements.

The long-term stability of the NPs stored at 4 °C in the refrigerator was also assessed in order to estimate the optimal usage time of the obtained colloidal suspensions. The average hydrodynamic diameter of the NPs was determined and is presented as a mean value of the three independent measurements.

#### 2.3.3 Atomic Force Microscopy (AFM)

Atomic force microscopy (AFM) studies were performed using a Dimension Icon AFM (Bruker, Santa Barbara, CA, USA) working in the PeakForce QNM mode. Suspension of SPION/HA and SPION/HA-FA (0.1 mg/mL) was deposited on a silicon plate using a spin-coating method. The silicon plates were preconditioned by cleaning with ethanol and Ultra-Violet Ozone Cleaner and dip-coating with a thin layer of poly(ethylenimine) (1 mg/mL aqueous solution) to facilitate the electrostatic attachment of the nanoparticles on the surface. AFM measurements were performed in air using ScanAsyst-Air probes (nominal spring constant, 0.4 N/m) or in deionized water using ScanAsyst-Fluid+ probes (nominal spring constant, 0.7 N/m). The AFM topography maps were acquired with a total resolution of 256 × 256 pixels using low loading forces to prevent sample damage. During drying, the nanoparticles formed agglomerates, and their polymer cores were entangled, making size analysis difficult. Therefore, the size of nonagglomerated or those with easily distinguishable boundaries was measured using Nanoscope 3.0 particle analysis software. Eight particles were selected each time to estimate the average size.

#### 2.3.4 TEM and Cryo-TEM

TEM imaging was carried out with a Tecnai Osiris instrument (FEI) with an X-FEG Schottky field emitter operating at an accelerating voltage of 200 kV and a Rio16 camera. The samples were deposited on a lacey carbon film supported on a copper grid (Agar Scientific, 400 mesh).

The SPION samples for cryo-TEM imaging were prepared with a Vitrobot Mark IV (Thermo Fisher Scientific). Three microliters of the sample were placed on the microscope grid, the excess was extracted with filter paper, and the slide was immersed in liquid ethane at high speed, leading to the entrapment of SPION nanoparticles in amorphous ice (vitrification parameters used: blot force: 4, blot time: 2 s, waiting time: 30 s). The measurements were performed using a Thermo Scientific Glacios Cryo Transmission Electron Microscope (Cryo-TEM) at an accelerating voltage of 200 kV.

#### 2.3.5 X-ray Diffractometry (XRD)

X-ray diffractometry (XRD) analysis was performed at room temperature with a Siemens D5000 diffractometer (Siemens, München, Germany) equipped with a copper X-ray tube. The Cu Kα radiation was used in the measurements. Samples were prepared for measurement by drying the colloidal dispersion of the nanoparticles on a zero-diffraction silicon sample holder. The analysis of the diffractograms was conducted with the X’Pert Highscore Plus software.

#### 2.3.6 ATR-FTIR

The chemical structure of the obtained SPIONs was determined by ATR-FTIR spectroscopy using a Thermo Fisher FTIR Nicolet iS10 spectrometer with the attenuated total reflectance (ATR) accessory (Thermo-Fisher Scientific, Warsaw, Poland). Data were recorded and analyzed using OMNIC FTIR software.

#### 2.3.7 Magnetometry

Determination of magnetic properties was performed with a Vibrating Sample Magnetometer option of a Quantum Design Physical Property Measurement System (PPMS) (San Diego, USA) equipped with a 9 T superconducting magnet.

#### 2.3.8 Mössbauer Spectroscopy

^57^Fe Mössbauer measurements were carried out at room temperature in transmission mode with a constant acceleration spectrometer (Elektronika Jądrowa, Cracow, Poland). A source of 5 mCi ^57^Co in a rhodium matrix was used.

#### 2.3.9 Nuclear Magnetic Resonance

For the nuclear magnetic resonance measurements, a PS-15 spectrometer manufactured by Ellab (Poznań, Poland), operating at a frequency of 15 MHz (0.35 T) was used. The ^1^H relaxation times T1 and T2 were measured at room temperature. The inversion–recovery (IR) and the Carr–Purcell–Meiboom–Gill (CPMG) sequence was applied.

#### 2.3.10 Magnetic Resonance Imaging (MRI)

MRI scans were performed at the 0.6 T Magritek MRI system at the Faculty of Geology, Geophysics, and Environmental Protection of the AGH University of Kraków (courtesy of Prof. Artur Krzyżak). Imaging was performed on a phantom constructed of Eppendorf tubes filled with SPION/HA or SPION/HA-FA colloidal aqueous suspensions evenly distributed in 2% agarose gel.^28,29^ The nanoparticles’ samples with Fe concentrations in the range of 0–0.5 mM Fe were used. The spin–echo sequence at the repetition time of 10 s and the spin–echo time of 7 ms was used for a T2-weighted scan.

### 2.4 Biocompatibility Assessment

#### 2.4.1 Hemolytic Activity of Nanoparticles

Human blood from healthy volunteers came from the Regional Center for Blood Donation and Treatment in Krakow (Poland). The Regional Center of Blood Donation and Treatment deidentified blood materials as appropriate for the confidentiality of human subjects. Thus, this study adheres to relevant exclusions from the approval of human subjects. Human blood samples were diluted with PBS to obtain a total hemoglobin concentration of up to 10 mg/mL and then used to evaluate the hemolytic activity of the nanoparticles. Samples with a final volume of 0.5 mL were prepared as follows: 0.05 mL of diluted human blood was mixed with 0.4 or 0.282 mL of PBS and with (a) 0.05 mL of PBS (negative control—untreated erythrocytes), (b) 0.118 mL of SPION/HA, (c) 0.05 mL of SPION/HA-FA, or (d) 0.05 mL of 4% Triton-X100 (positive control—complete release of hemoglobin). The volume of SPION nanoparticles was calculated based on the amount of iron ions (Fe), which finally amounted to 53.1 μg/mL. In addition, samples without diluted human blood were prepared to control the absorbance value of SPION nanoparticles. After 3 h incubation at 37 °C, the erythrocytes were centrifuged (1500 g, 5 min), and the hemolytic activity of the nanoparticles was measured spectrophotometrically at 540 nm (Synergy H1 hybrid plate reader and Gene 5 version 2.00.18 Software (BIOTEK Instruments, Winooski, VT, USA) after performing the Drabkin reaction, as previously described.^30^ The experiment was repeated twice using blood obtained from two different volunteers.

#### 2.4.2 Cell Viability Using Luminescence ATP Detection Assay System

Human peripheral blood mononuclear cells (hPBMC) and human neutrophils (hPMN) were isolated from the blood using a method based on centrifugation in a Ficoll-Paque Plus density gradient (Ge Healthcare, Chicago, IL, USA), and poly(vinyl alcohol) (POCH, Gliwice, Poland) was applied to eliminate erythrocytes. hPBMC and hPMN were used in experiments immediately after isolation. To analyze the toxicity of tested nanoparticles, hPBMC (1 × 10^6^ cells/mL) and hPMN (1 × 10^6^ cells/mL) were transferred to the wells of a 96-well plate in 100 μL of RPMI (Lonza, Basel, Switzerland) supplemented with 20% (hPBMC) or 10% (hPMN) fetal bovine serum (v/v) (FBS, GIBCO, Paisley, U.K.) and antibiotics (penicillin at concentration 100 U/mL and streptomycin at concentration 100 μg/mL, GIBCO, Paisley, U.K.). Then, SPION/HA or SPION/HA-FA was added to the cells at final concentrations of 5, 10, or 25 μg/mL (based on the amount of Fe). Cell viability was analyzed after 24 h of incubation using an ATPlite kit using the manufacturer’s procedure (PerkinElmer, Waltham, MA, USA). Chemiluminescence was measured using a Synergy H1 hybrid plate reader and Gene 5 version 2.00.18 Software (BIOTEK Instruments, Winooski, VT, USA).

#### 2.4.3 HepG2 Viability Using MTT Assay

Human hepatoma-derived HepG2 cells (ATCC HB-8065) obtained from the American Type Culture Collection (Manassas, VA, USA) were grown in DMEM GlutaMAXTM medium containing 1 g/L of 10% (v/v) glucose and 10% (v/v) FBS and antibiotics (all these reagents were from GIBCO, Paisley, U.K.). For the experiment, the cells were trypsinized (Lonza, Basel, Switzerland), centrifuged, and transferred to the wells of a 96-well plate in 100 μL/well (at the density of 5 × 10^4^ cells/mL) of medium containing 10% (v/v) of FBS and antibiotics. After overnight growing, SPION/HA or SPION/HA-FA was added to the cells for 24 h. The final Fe concentration was 5, 10, or 25 μg/mL. The toxicity of the tested nanoparticles was analyzed using the MTT test and standard procedure (Sigma, St. Louis, MO, USA). The absorbance was measured at 545 nm using a Synergy H1 hybrid plate reader and Gene 5 version 2.00.18 Software.

### 2.5 Studies on the Human A172 Glioblastoma Multiforme Cell Line and Human Adenocarcinoma SW620 Cell Line

#### 2.5.1 A172 and SW620 Cell Viability by MTT Assay

The human glioblastoma multiforme A172 cell line (ATCC) and human adenocarcinoma SW620 cell line (ATCC) were cultured in a high-glucose Dulbecco’s modified Eagle’s (DMEM) medium, supplemented with 10% (v/v) FBS and 1% penicillin–streptomycin at 37 °C and 5% CO_2_. Cells were subcultured until the cells reached 80% confluence, and next, they were trypsinized and seeded on sterile 96-well plates (2.0 × 10^4^ cells/well for A172 cells and 3.0 × 10^4^ cells/well for SW620 cells, respectively).

The cytotoxic activity of SPION/HA and surface-functionalized SPION/HA-FA was assessed using the MTT test and standard procedure (Sigma, St. Louis, MO, USA). Briefly, cells were seeded in 96-well culture plates, and after 24 h, medium was replaced with 100 μL of the fresh one containing different concentrations of obtained SPIONs. After 24, 48, and 72 h, respectively, 5 μL of MTT solution (5 mg/mL in PBS) was added into each well and cell culture plate was incubated for 3 h under culture conditions. The obtained purple formazan precipitate was dissolved using a solubilization buffer (50 μL; 10% SDS in 0.01 M HCl), and the plates were left overnight in culture conditions. The absorbance was measured at 570 and 690 nm (background) using an ELISA plate reader (Synergy HT, BIO-TEK, USA). Each result is presented as a mean of the two independent experiments, each of which was performed in triplicate. For tests with the NPs, the signal from SPIONs alone was detected as a blank and subtracted.

#### 2.5.2 Cellular Uptake

SPION uptake by human A172 glioblastoma multiforme cells was studied by confocal microscopy. Cells were seeded onto glass coverslips placed in a 6-well cell culture plate at (2 × 10^5^) cells/well and incubated for 48 h and then stimulated with FITC-labeled SPION/HA or SPION/HA-FA nanoparticles in PBS (pH = 7.4). The Fe concentration in each sample was about 10 μg/mL for SPION/HA and about 5 μg/mL for SPION/HA-FA. After stimulation, cells were incubated for 6 h under culture conditions, washed with PBS, and fixed by incubation in 4% formaldehyde solution (20 min at room temperature) along with permeabilization with 0.5% Triton X-100 solution (10 min at room temperature). The cells were then washed with PBS and the phalloidin-CF633 conjugate, and Hoechst 33342 working solutions were used to stain F-actin filaments and cell nuclei, respectively. The samples were then visualized using a Nikon Ti-E inverted microscope with a Nikon A1 confocal system and 405, 488, and 638 nm diode lasers for excitation. Images were obtained in emission mode using 40 or 100× oil objectives.

## 3 Results and Discussion

### 3.1 Synthesis and Physiochemical Characterization of the SPION/HA

HA-coated SPIONs were synthesized based on a few literature reports, concerning the synthesis of iron oxide nanoparticles stabilized by various polysaccharides, e.g., dextran, as presented by Schemberg et al.,^31^ or chitosan, as reported by Szpak et al.^24^ Reaction conditions, such as HA and iron salt concentrations, reaction time, temperature, pH, and type of the base used for precipitation, were optimized in order to obtain SPION/HA nanoparticles of a desired size, size distribution, colloidal stability, and magnetic properties. The proposed synthesis yielded desired nanoparticles in a one-step process, with a stable HA coating forming on the surface of iron oxide cores directly during the coprecipitation reaction.

Na-HA solution was first sonicated for 3 h in 50 °C^32^ in the presence of ammonia to facilitate the breakdown of supramolecular interactions of HA chains in aqueous solution.^33−35^ The introduction of iron salts initiated the coprecipitation process and formation of the magnetic nanoparticles. A further addition of another 4 mL of ammonia solution ensured that the pH value of the mixture was above 10.^36^ The reaction lasted 30 min, what allowed for the formation of a stable colloidal suspension of SPION/HA.^24^ The synthesis was carried out under relatively mild conditions compared with procedures described previously. For example, Zhang et al.^20^ obtained similar particles by coating previously synthesized SPIONs with HA. The process was carried out at 80 °C for 2 h. On the other hand, Soleymani et al.^21^ obtained HA-coated SPIONs in a one-step process, but the reaction took 24 h and required a much higher temperature (150 °C).

Based on the DLS measurements, the average hydrodynamic diameter of the obtained SPION/HA (see Table 1 and Figure 6A), was 137 nm, with a zeta potential greater than −50 mV, indicating an excellent colloidal stability of the obtained nanoparticles.

**Table 1:** Size, PDI, and Zeta Potential of Obtained SPION/HA and SPION/HA-FA Based on DLS Measurements

type of the SPIONs	DLS measurements		
	Z-Av (nm)	PDI	zeta potential (mV)
SPION/HA	137 ± 2	0.26 ± 0.01	–53 ± 1
SPION/HA-FA	182 ± 2	0.20 ± 0.01	–49 ± 1

**Figure 2:**
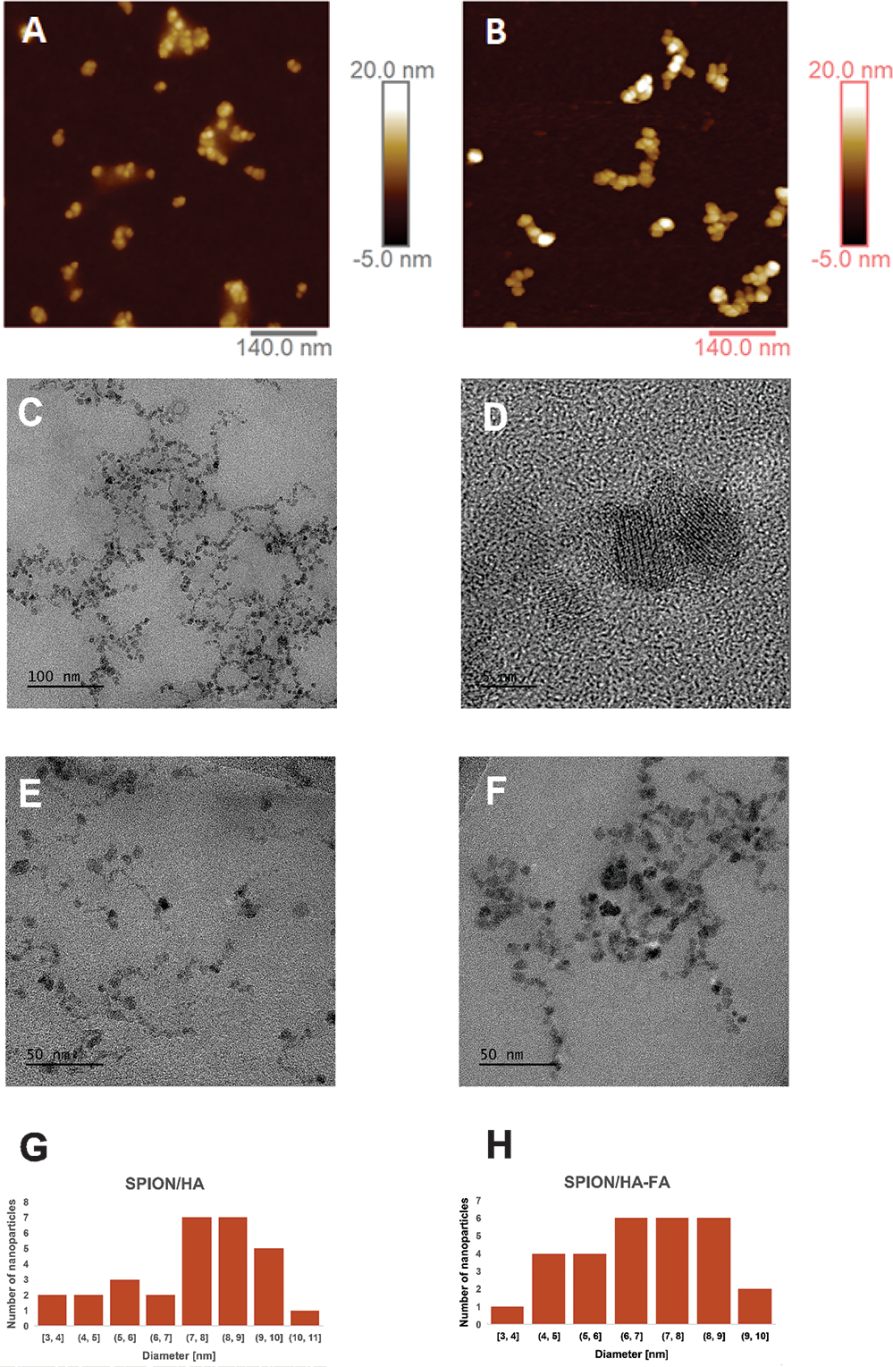
AFM images of (A) SPION/HA and (B) SPION/HA-FA. TEM images (C,D) of SPION/HA-FA (×64,000 and ×930,000). Comparison of (E) SPION/HA and (F) SPION/HA-FA at the same scale (×130 000). (G) Histogram of SPION/HA and (H) histogram of SPION/HA-FA.

The morphology of the synthesized SPIONs was studied using AFM and cryo-TEM. The AFM images obtained on air (dry samples) are presented in Figure 2. The obtained nanoparticles are spherical and after drying show a tendency to form bigger agglomerates outflanked with HA chains (see Figure 2A). The mean diameter of dry SPION/HA, based on the performed measurements, was about (20 ± 2) nm. Polymeric coating of SPION/HA swells considerably in aqueous media; thus, the AFM measurements were also performed in water. Various loading forces, ranging from 0.5 to 3 nN, were used to visualize the significant swelling of the hyaluronic acid present on the surface of SPIONs (Supporting Information Figure S1). The relatively large difference observed in the diameter values of the dry and wet nanoparticles was due to a combination of two effects: the presence of an additional hydration layer and significant swelling of the HA coating in aqueous media. The latter was further explored and detailed in Supporting Information.

TEM images further confirmed the spherical shape of the obtained nanoparticles. Iron oxide cores of ca. 6–7 nm are embedded within the thin HA coating. The average size of the dry magnetic nanoparticles was estimated as (7.5 ± 2) nm. Although the nanoparticles tend to form clusters, resembling small chains of beads, they are visibly separated and no significant aggregation is observed.

**Figure 3:**
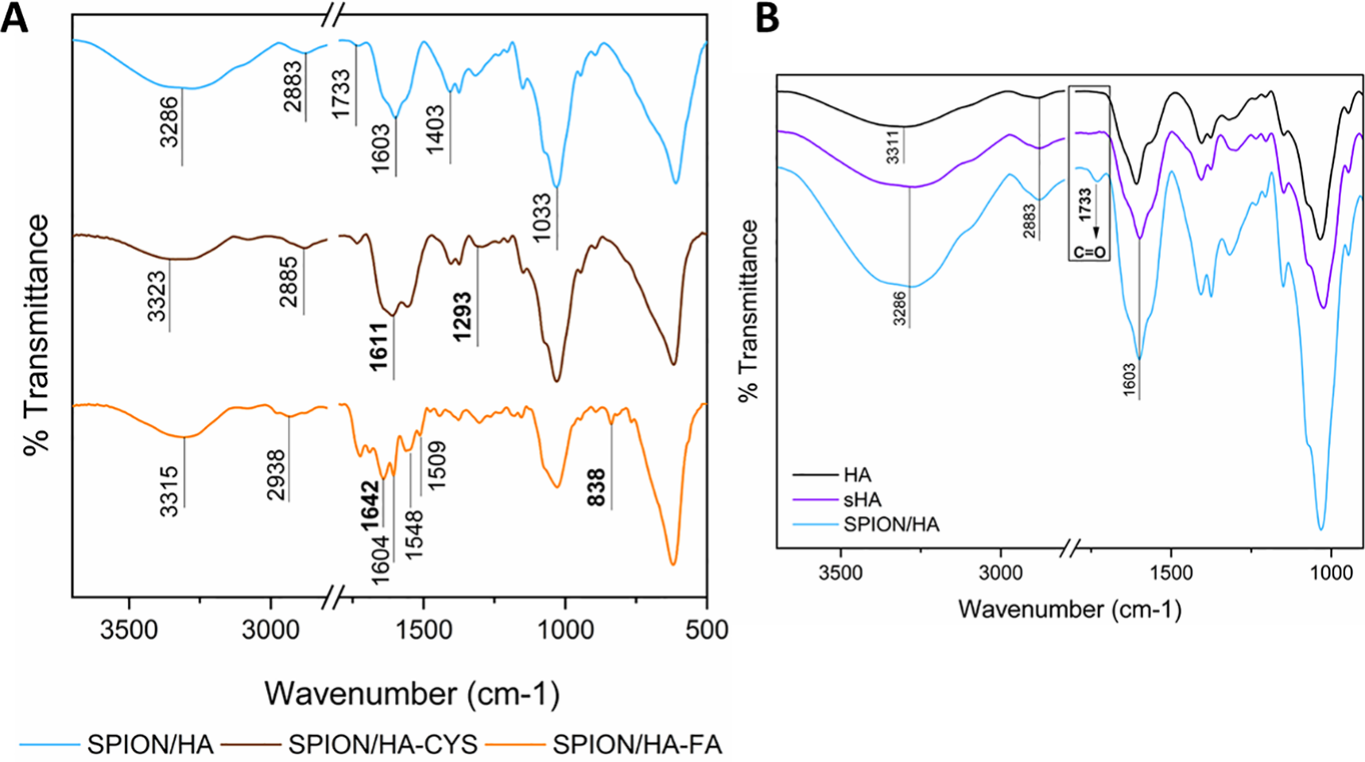
ATR-FTIR spectra of obtained (A) SPION/HA, SPION/HA-CYS, and/SPION/HA-FA and (B) HA, sHA, and SPION/HA.

The presence of hyaluronic acid on the surfaces of SPIONs was also confirmed by ATR-FTIR (Figure 3A). In comparison to bare SPIONs,^37^ the spectrum of the SPION/HA sample exhibited bands characteristic for HA coating at 3286 cm^–1^ (−OH stretching vibrations of the carboxylic groups), 2883 cm^–1^ (−CH stretching vibrations), and 1603 and 1403 cm^–1^ (−COO antisymmetric and symmetric stretching vibrations^38^). A new band appearing at 1733 cm^–1^ for SPION/HA can be assigned to the C=O vibration in aldehydes, pointing out to the possible partial oxidation of hydroxyl groups in glucuronic acid moiety.^39^ Taking it into consideration, HA was sonicated in the same conditions as during SPION/HA synthesis, freeze-dried (sHA) and characterized by ATR-FTIR (see Figure 3B) and NMR (Supporting Information, Figure S2) to assess, on which step of the synthesis, the possible oxidation might occur.

As could be seen in Figure 3B, the ATR-FTIR spectrum of sHA does not exhibit the specific band at 1733 cm^–1^. Also, the NMR spectra of HA and sHA do not show differences, suggesting that the oxidation must have occurred during the main synthetic step, which was the precipitation of SPIONs. The oxidized HA hydrogels are, however, known as biocompatible drug carriers with better injectability.^40^ Thus, partial oxidation of the HA coating may be beneficial for the fabrication of our nanoparticulate systems.

**Figure 4:**
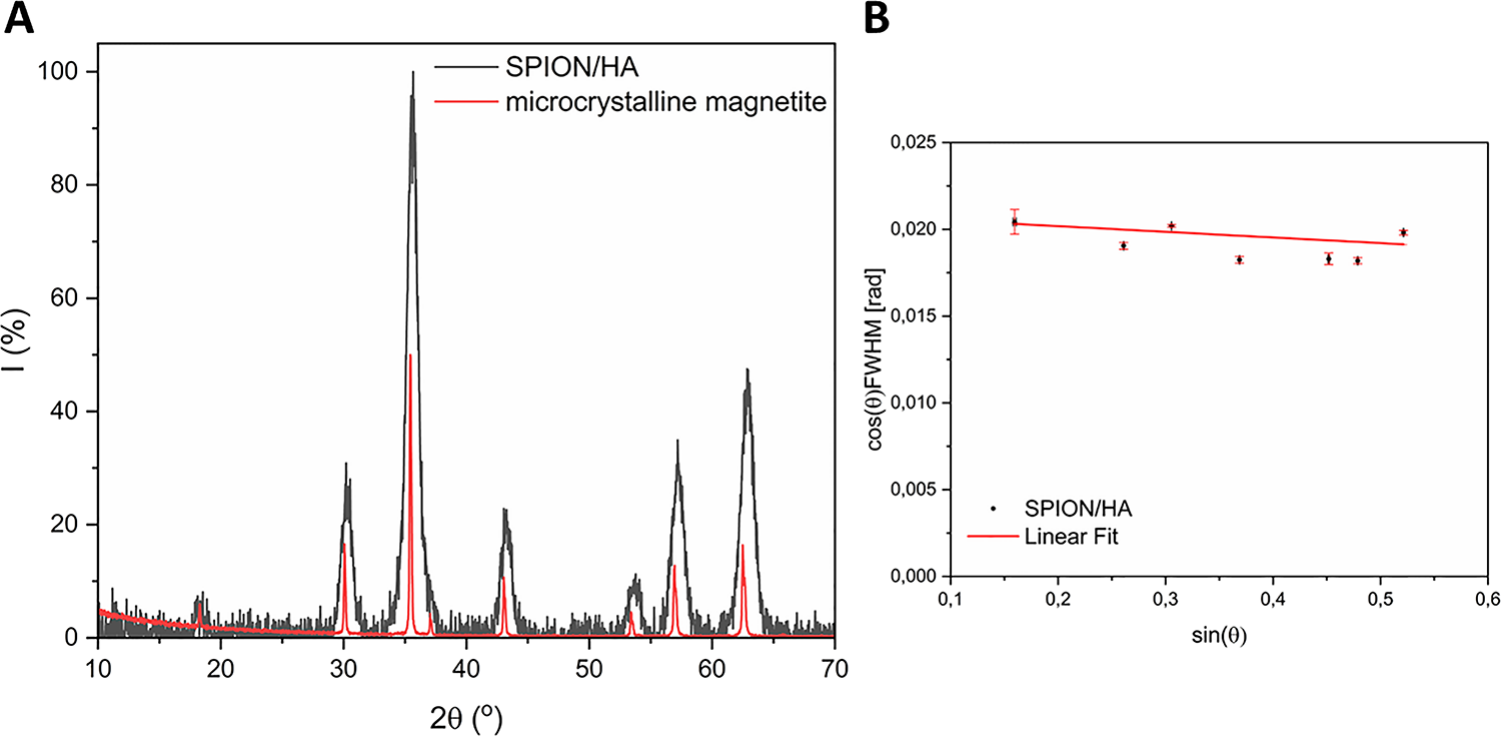
(A) Diffractograms of SPION/HA and microcrystalline magnetite (magnetite lines’ intensity is scaled down by the factor of 2 for clarity) and (B) Williamson–Hall plot of the sample (fitted parameters obtained are slope = −0.003, intercept = 0.021).

Diffractogram of HA-coated SPIONs was also obtained, and the results are plotted in Figure 4A, along with a diffractogram of microcrystalline magnetite. The lines in the pattern of the sample correspond to those of the microcrystalline magnetite. However, a considerable line broadening is noted in comparison with the microcrystalline magnetite, along with a slight shift to higher angular values. The line broadening is due to the nanoparticulate character of the sample. The shift of the lines to the higher values may be explained by the smaller lattice constants compared to magnetite, which suggests the presence of maghemite in the magnetic cores of the SPION/HA. Maghemite is an oxidized form of magnetite, which realizes through cation vacancies.^41^

Lorentzian profiles were fitted to the Bragg peaks and provided their half-width values. Then, Williamson–Hall plots were constructed (see Figure 4B), and the mean diameters of the magnetic cores of SPION/HA were derived from linear fits as about 7 nm.

### 3.2 Magnetic Properties of the SPION/HA

**Figure 5:**
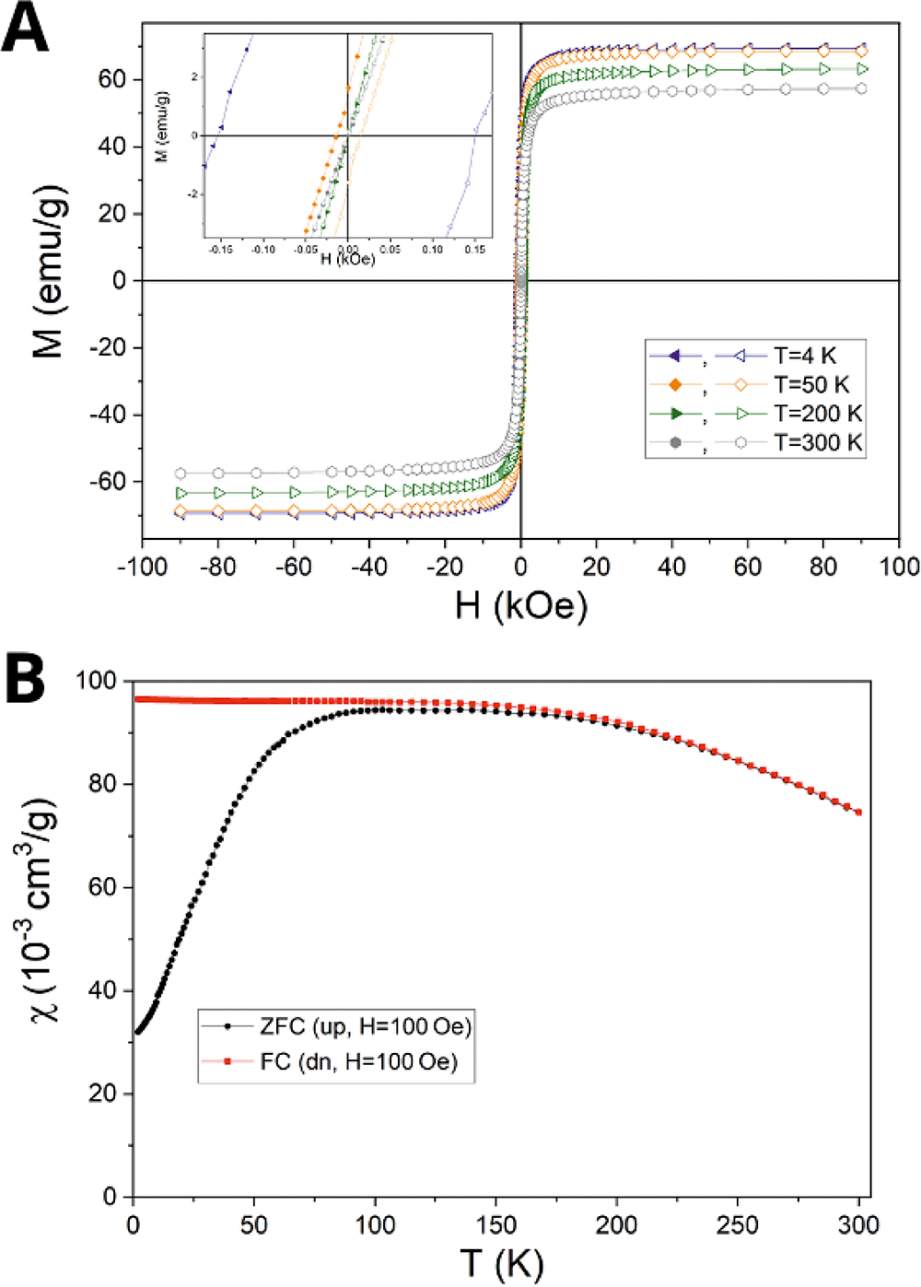
(A) Full plot of the magnetization of the SPION/HA in the range of temperatures between 4 and 300 K (hysteresis loops); the inset shows the hysteresis loops zoomed at the origin, (B) magnetic susceptibility of the SPION/HA, with zero field cooling curve (black) and field cooling curve at 100 Oe (red).

Magnetometric measurements provided the magnetization curves (Figure 5A) and the susceptibility curves (Figure 5B) obtained at various temperatures.

The magnetic saturation was achieved for a magnetic field strength of 20 kOe (Figure 5A). The coercive field is 180 Oe at 2 K and gradually decreases with increasing temperature to reach zero at 100 K, where the hysteresis disappears, as can be seen in Figure 5A-inset. This is consistent with the observed behavior of the zero-field-cooled (ZFC) and field-cooled (FC) low field magnetic susceptibilities (Figure 5B) which have their temperature dependences characteristic for a superparamagnet. The superparamagnetic blocking temperature corresponds in our case to 100 K. Above this temperature the magnetic moments of nanoparticles fluctuate due to thermal excitation, which corresponds to the superparamagnetic fluctuations state. The vanishing hysteresis in the magnetization loops (Figure 5B) is an indicator of such a state. The ZFC and FC magnetic susceptibility curves diverge already above 100 K which indicates a distribution of blocking temperatures.

The values of coercive field, saturation magnetization, and remanence are collected in Table S1 (see Supporting Information), and their temperature dependences are plotted in Figure S5 (see Supporting Information). The same coercivity values at the negative and positive field denote a lack of the exchange bias effect. The saturation magnetization value at room temperature amounts to 57 emu/g and increases to 69 emu/g on decreasing the temperature to 4 K. The Mössbauer spectroscopy study was performed for the SPION/HA. The Mössbauer spectrum obtained at room temperature is presented in Figure S6 (see Supporting Information) and contains a heavily broadened single line with the shape characteristic for the relaxational spectra of superparamagnetic materials.

Magnetic nanoparticles are usually considered as T2 contrast agents for MRI because they produce fluctuating local magnetic field inhomogeneities. However, since they have been observed to exhibit magnetic fluctuation frequencies in a range that overlaps with scanner frequencies (typically ∼10–130 MHz), they can also produce significant T1 contrast (dual modal Gd-containing SPION contrast agents^42^). That happens when the frequency of magnetic fluctuations is close to the Larmor frequency of nuclear spins.

### 3.3 Synthesis and Physiochemical Characterization of the SPION/HA-FA

As FA can only actively target its receptor with free -NH_2_ end,^43^ this would not be possible, if FA was coupled directly with carboxylic groups of hyaluronic acid of SPION/HA. The proposed surface functionalization of SPION/HA consisted of two steps (see Figure 1), where in the first one, the free amino groups were introduced by CYS coupling. Reaction conditions were established based on the report by Yang et at.^25^ The presence of CYS on the NPs’ surface was confirmed by ATR-FTIR (Figure 3A), where the characteristic bands were observed: the N–H stretching vibrations of primary amines at 1611 cm^–1^ and C–N stretching vibrations of primary amines at 1211 cm^–1^. The amount of the primary amino groups on the surface of SPION/HA-CYS was assessed spectrophotometrically by TNBS method,^25^ where the concentration of the free -NH_2_ groups was estimated as (2.2 ± 0.1) mM.

**Figure 6:**
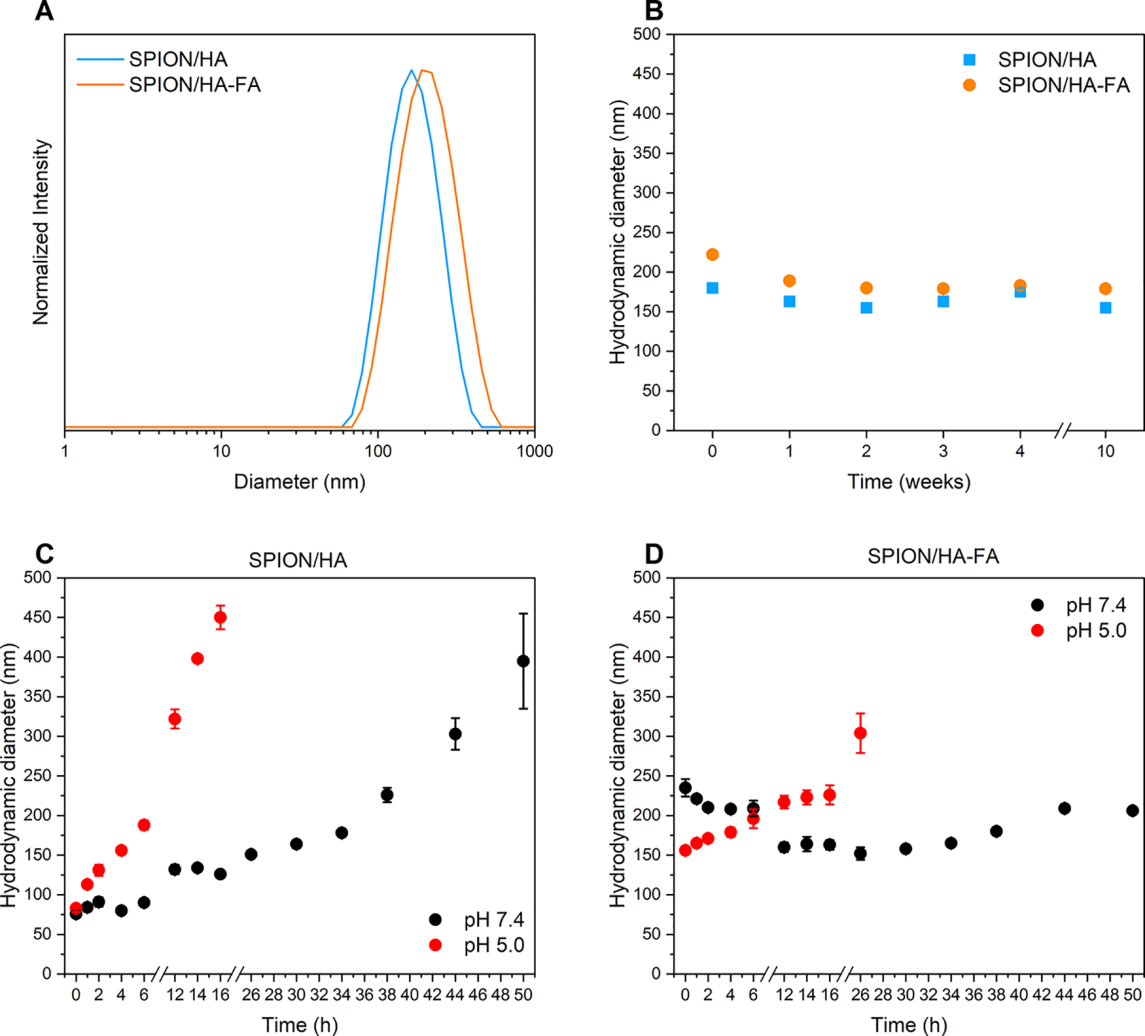
(A) DLS size distribution diagrams of SPION/HA and SPION/HA-FA. Results are presented as mean ± SD (*n* = 5). (B) Long-term changes in the hydrodynamic diameter of SPION/HA and SPION/HA-FA at the storage conditions (refrigerator at 4 °C) with the storage time. (C) Changes in the hydrodynamic diameter of SPION/HA incubated at 37 °C and pH = 7.4 or pH = 5.0. (D) Changes in the hydrodynamic diameter of SPION/HA-FA incubated at 37 °C and pH = 7.4 or pH = 5.0.

The sizes of the obtained SPION/HA-FA nanoparticles were ca. 182 nm based on DLS measurements (see Table 1 and Figure 6A); thus, they were predictably larger than SPION/HA. Zeta potential of the NPs was slightly higher after the functionalization process yet still about −48 mV, which suggests the excellent colloidal stability of the FA-modified nanoparticles. Moreover, PDI value dropped to about 0.20, indicating low dispersity of SPION/HA-FA. Maintaining a high absolute zeta potential while reducing PDI was a favorable outcome of the functionalization process.

The morphology of the obtained NPs was studied using AFM in the same conditions (partially swollen sample) as for SPION/HA. As shown in Figure 2B, SPION/HA-FA particles are bigger than SPION/HA before surface functionalization (Figure 2A) and tend to form agglomerates when dry. However, those agglomerates are not visibly embordered by HA as in the case of SPION/HA, suggesting that introducing of CYS and FA impacted the behavior of the hyaluronic coating. Based on AFM, the mean diameter value for partially swollen SPION/HA-FA was (26 ± 4) nm. The average hydrodynamic diameter of both SPION/HA and SPION/HA-FA was thus well below 200 nm, in a range suitable for both passive and active targeting of cancer cells—the latter through specific interactions of the particles with hyaluronic acid and/or folic acid receptors.^44−46^ This was also confirmed in the uptake studies using confocal microscopy (see Section 3.6.2), where both types of nanoparticles successfully entered the A172 cells tested.

TEM images of SPION/HA-FA do not differ significantly from the ones observed for SPION/HA. They are small, with the core of ca. 6–7 nm and very thin polymeric coating, and form small clusters resembling the chains of beads. Their average size is somewhat smaller (6.9 ± 2) nm compared to SPION/HA, suggesting that the attachment of FA slightly limited the swelling ability of HA. Based on cryo-TEM study (Supporting Information, Figure S3), the swollen SPION/HA-FA nanoparticles were visibly larger, with their average size in the range of 25–35 nm, which corresponds well with AFM results.

ATR-FTIR spectrum of SPION/HA-FA (see Figure 3A) exhibits the bands characteristic for the amide bond formation: amide I at 1642 cm^–1^ (governed mainly by the stretching vibrations of the C=O and C–N groups) and amide II at 1548 cm^–1^ (mainly in-plane N–H bending). The presence of those bands, as well as the band 838 cm^–1^ (aryl-H deformation vibration in aromatic ring), originating from FA’s structure, suggests that FA was successfully attached to the functionalized SPIONs. FA content was also assessed spectrophotometrically (see Supporting Information, Figure S4). The maximum at 360 nm characteristic for FA was present only in the spectrum of SPION/HA-FA. Based on the spectrophotometric analysis, the percentage of the FA present on the surface of the nanoparticles was estimated as (28.5 ± 2.9) %.

Long-term stability of the obtained colloidal suspensions was also assessed. The observed by TEM and AFM clusters of both types of the NPs remained stable for over 10 weeks, when stored at 4 °C (see Figure 6B). The observed mean hydrodynamic diameter at week 10 was (155 ± 2) nm with PDI value of (0.20 ± 0.02) for SPION/HA, whereas for SPION/HA-FA, these values were (179 ± 2) nm and (0.22 ± 0.01), respectively. Thus, the size of SPION/HA changed by only 11% within 10 weeks, while for FA-bearing NPs, no change was observed within the scope of statistical error. The colloidal stability of the SPIONs in simulated physiological conditions was also assessed in conditions characteristic for both the blood/healthy tissues (PBS of pH = 7.4) and the tumor site (PBS of pH = 5.0) (see Figure 6C,D). The initial values of the hydrodynamic diameters of SPION/HA and SPION/HA-FA at pH = 7.4 were (76 ± 5) nm and (235 ± 11), respectively, whereas at pH = 5.0, they were (83 ± 1) for SPION/HA and (156 ± 5) for SPION/HA-FA. These results suggest that, as expected, the hydrogel coating of our particles is significantly influenced by pH and ionic strength. The colloidal stability of SPION/HA was visibly influenced by the pH of the buffer solution, as a steady increase in the hydrodynamic diameter value was observed upon incubation in PBS in both pH = 7.4 and pH = 5 (see Figure 6C). At pH = 7.4, the size of SPION/HA did not change drastically, and their size was still below 200 nm up to 34 h, so despite the slow increase in diameter, they may be considered relatively stable. At pH = 5, SPION/HA are not stable, although for the first 6 h, the changes in their size are slower and their size is still below 200 nm. The surface modification with FA considerably increased the colloidal stability of the nanoparticles (see Figure 6D). SPION/HA-FA remained stable for 50 h at pH = 7.4 and for 16 h at pH = 5.0.

### 3.4 Evaluation of the NMR Relaxation Rates for SPION/HA and SPION/HA-FA

For NMR measurements, CPMG sequence was used to determine the T2 and the IR sequence was utilized to determine T1. The samples were diluted in distilled water to obtain desired concentrations for studying the relationship between the concentration and the relaxation rates (1/T1 and 1/T2).

**Figure 7:**
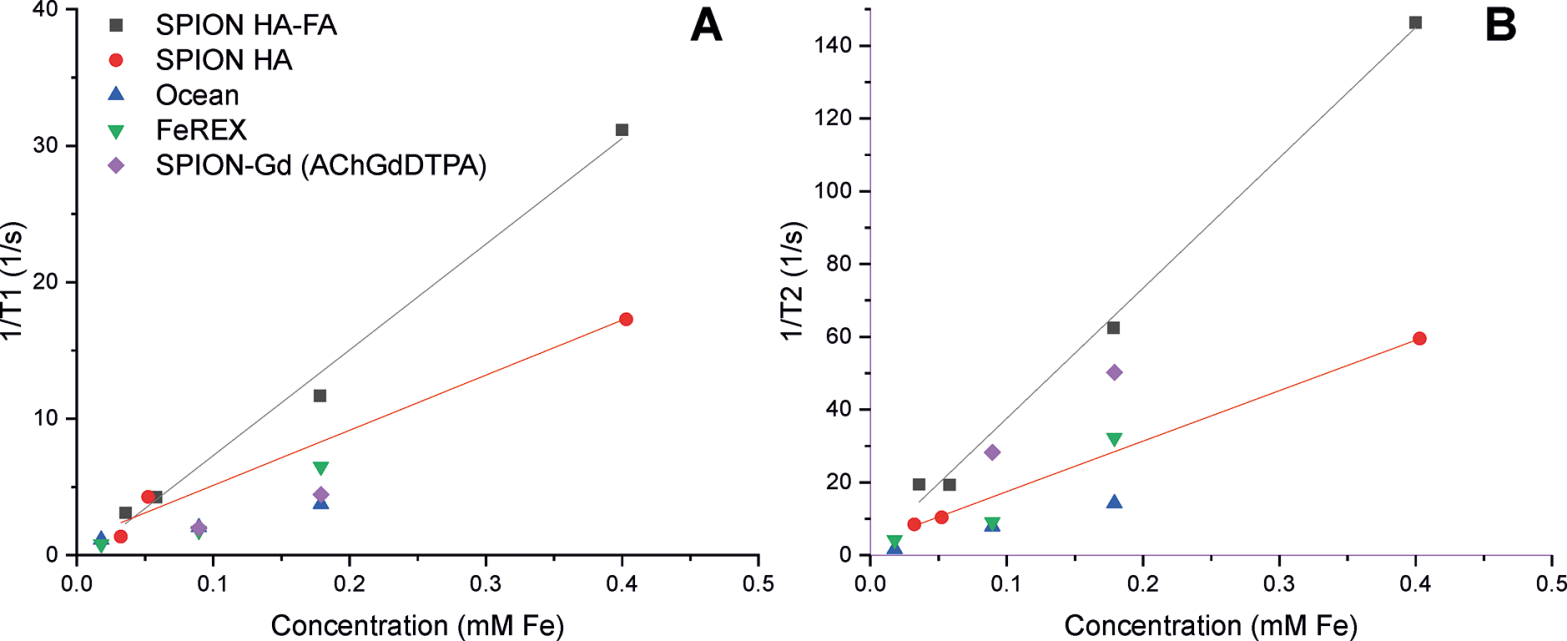
(A) ^1^H NMR relaxation rates 1/T1 for SPION/HA and SPION/HA-FA versus their concentration expressed in mM of Fe (literature data [Szpak et al.^42^] is added for a comparison) and (B) ^1^H NMR relaxation rates 1/T2 for SPION/HA and SPION/HA-FA versus their concentration expressed in mM of Fe (literature data [Szpak et al.^42^] are added for a comparison).

Figure 7 shows the dependence of the relaxation rates 1/T1 (Figure 7A) and 1/T2 (Figure 7B) on the concentration of iron in the SPION suspensions. It can be noticed that the slope (r2(T2)) for the FA-functionalized nanoparticles, amounting to 358 L/(mmol s), is more than twice the value for the nonfunctionalized nanoparticles (139 L/(mmol s)). Consequently, SPION/HA-FA will have much higher effectiveness as a T2 contrast agent in comparison with SPION/HA.

### 3.5 Magnetic Resonance Imaging (MRI)

**Figure 8:**
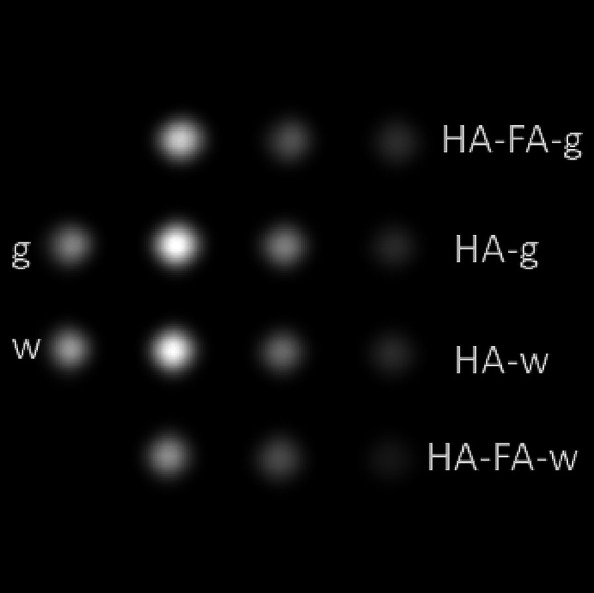
T2-weighted (repetition time 10 s, echo time 7 ms) image of MRI scan of Eppendorf tubes containing different concentrations (mM Fe, see column labels) of SPION/HA and SPION/HA-FA water suspensions (w) and gel phantoms (g), together with the reference samples of water (w) and gel (g).

For MRI measurement, the phantoms constructed using Eppendorf tubes (inner diameter of 6 mm) were used. They were filled with SPION/HA or SPION/HA-FA suspensions in 2% agarose gel of different concentrations. The suspensions of the nanoparticles in water in the same concentrations were measured for a comparison, along with water and gel samples with no SPIONs. The T2-weighted image obtained for a cross-section of the phantom is presented in Figure 8.

The measurements carried out showed the high effectiveness of SPION/HA and SPION/HA-FA materials as contrast agents for the T2 relaxation time-weighted scan. The signal (brightness) decreases with increasing concentration for all four sample sets, although the decrease is more pronounced for SPION/HA-FA than for SPION/HA. This is consistent with a higher T2 relaxivity obtained for SPION/HA-FA in the NMR measurements. Thus, the results obtained for the phantom indicate the high efficacy of our SPIONs as contrast agent for T2 relaxation time, confirming the potential of our SPIONs in MRI imaging of human tissues^47,48^

### 3.6 Biocompatibility Assessment of SPION/HA and SPION/HA-FA

#### 3.6.1 Hemolytic Activity of Nanoparticles

**Figure 9:**
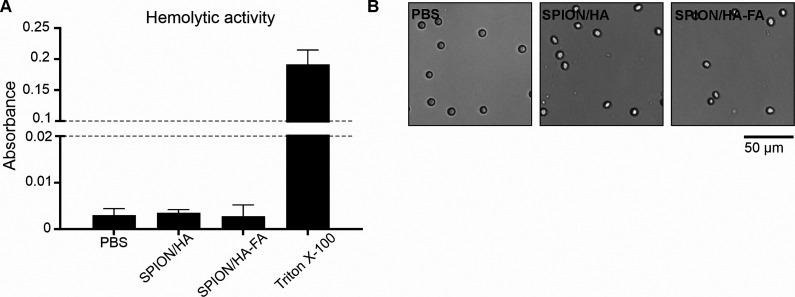
Measurement of the hemolytic activity of SPION/HA and SPION/HA-FA. Human erythrocytes were incubated for 3 h with PBS (negative control of hemolysis), 0.4% Triton X-100 (positive control of hemolysis), or nanoparticles (SPION/HA or SPION/HA-FA). (A) Absorbance of the released hemoglobin was detected spectrophotometrically after reaction with Drabkin’s reagent. Bars represent mean ± SD from two different experiments. (B) Erythrocyte morphology for SPION/HA, SPION/HA-FA and the control in PBS.

Regardless of the route of administration, nanoparticles may interact with erythrocytes susceptible to hemolysis. The hemolytic activity of nanoparticles is presented as the absorbance of hemoglobin released from red blood cells after incubation with SPION/HA and SPION/HA-FA. As shown in Figure 9, none of the tested nanoparticles caused a hemoglobin leakage greater than the hemoglobin released spontaneously from untreated erythrocytes. In addition, there are no significant changes in the morphology of red blood cells after 3 h of incubation with both types of SPION nanoparticles. The obtained results indicate the blood compatibility of the tested nanoparticles.

#### 3.6.2 Influence of the SPIONs on Human PBMC and Neutrophils: Hepatotoxicity Studies

**Figure 10:**
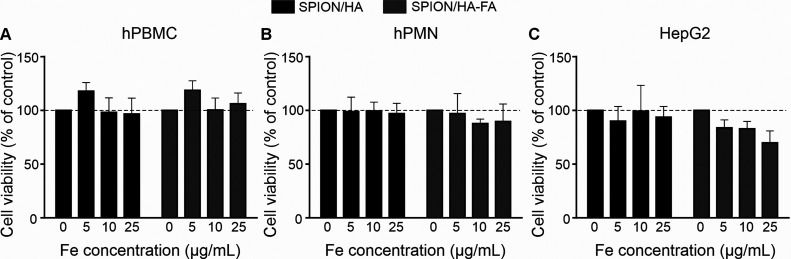
Analysis of SPION/HA or SPION/HA-FA interactions with human immune (hPBMC and hPMN) and liver (HepG2) cells. Cells were cultured in growth media containing PBS (control cells) or various doses of tested nanoparticles for 24 h. Viability of the cells (A) hPBMC, (B) hPMN was determined using ATPlite, and (C) HepG2 were analyzed by MTT test. Bars represent the mean ± SD from three different experiments.

To further evaluate the possible influence of our nanoparticles on the morphotic blood elements, we studied their interaction with human peripheral blood monocytes (hPBMC) and human polymorphonuclear neutrophils (hPMN). The results are presented in Figure 10. For hPBMC, no toxicity of nanoparticles was observed; however, for hPMN treated with SPION/HA-FA, a slight decrease in viability was detected (Figure 10A,B, respectively). Some observations indicate that human neutrophils can express folic acid receptors.^49^ Thus, the observed toxicity of SPION/HA-FA might result from the higher accumulation of these nanoparticles in hPMN than an accumulation of SPION/HA. To further evaluate the possible hepatotoxic effect of nanoparticles, hepatocytes were used. As they are important cells participating in the biodistribution and removal of nanoparticles from the body, they might be particularly vulnerable to damage caused by nanoparticles. HepG2 cells are a suitable in vitro model system of human hepatocytes, commonly used in studies aiming to analyze in vitro the toxicity of various agents and nanomaterials.

The cells were exposed to different doses of SPION/HA or SPION/HA-FA for 24 h. As presented in Figure 10C, only SPION/HA-FA decreased the viability of HepG2 cells in a dose-dependent manner. The viability of HepG2 cells treated with the highest dose of SPION/HA-FA (25 μg/mL) decreased to ∼70% compared with the cells treated with nanoparticles functionalized only with HA or control cells exposed to the medium containing PBS. These results indicate that nanoparticles functionalized with FA were probably better internalized by the HepG2 cells than SPION/HA.

Since hepatocytes are particularly sensitive to high doses of iron, intense internalization of tested nanoparticles could result in increased ROS production followed by damage to intracellular structures and disturbances in cell signaling, leading to a decrease in cell function and viability.^50^ Our results suggest that HepG2 cells internalize more nanoparticles functionalized with FA than those without folic acid on their surface. Therefore, higher cytotoxicity is observed for SPION/HA-FA. Thus, while SPION/HA nanoparticles may be applied in all concentrations studied, for the FA-modified system, only concentrations up to 10 μg/mL can be safely used. Our observations are in line with some other published data. Recently, Ala Hameed et al. demonstrated that HepG2 is a reliable model for studying the therapeutic potential of nanomaterials functionalized with FA for the delivery of anticancer drugs.^51^ At the same time, conflicting results were published concerning the expression of CD44 on HepG2. Hong-You-Yang et al. indicated HepG2 as CD44-negative cells and observed lower delivery of HA-modified hydrogels with cytochrome c or photosensitizers to HepG2 compared to hydrogel delivery to CD44-positive A549 cells.^52^ On the contrary, Lin-Song Li et al. observed a high uptake of HA-modified gold nanoparticles loaded with doxorubicin and indicated HepG2 cells as cells highly expressing CD44.^53^ Similarly, Yang et al. delivered to HepG2 doxorubicin loaded radiosensitive theranostic nanocarriers containing hyaluronan.

However, a mild toxic effect toward hepatocytes was also observed for FA-functionalized nanoparticles; thus, lower concentrations of these particles, below 25 μg/mL, should be used.

### 3.7 Studies on Cancer Cell Lines: Glioblastoma A172 and Colon Adenocarcinoma SW620

#### 3.7.1 MTT Assay

**Figure 11:**
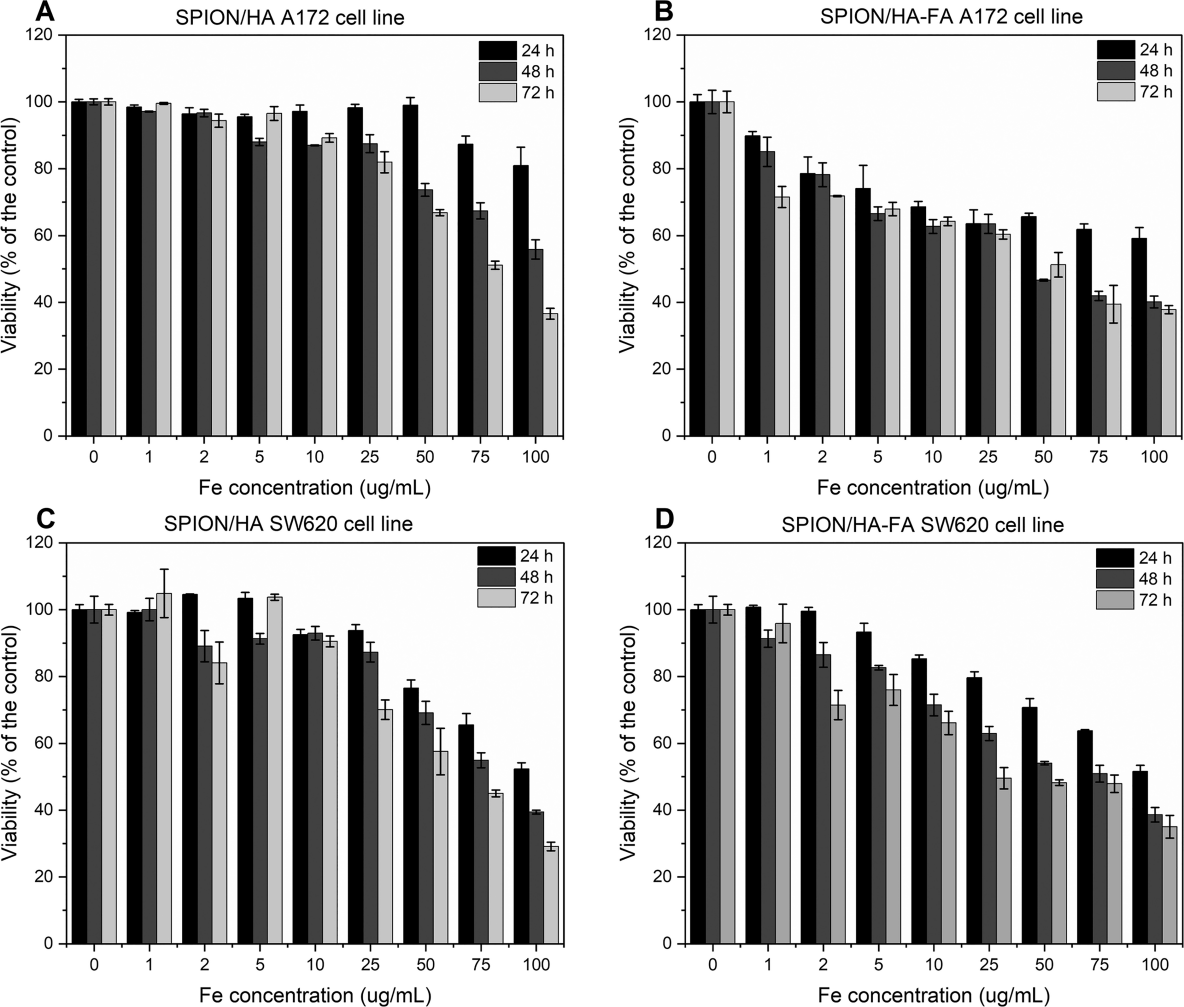
MTT test results for (A) A172 cells treated with SPION/HA, (B) A172 cells treated with SPION/HA-FA, (C) SW620 cells treated with SPION/HA, and (D) SW620 cells treated with SPION/HA-FA. Results are presented as mean ± SD (*n* = 3), as a percentage relative to the control.

The influence of SPION/HA and HASPION/HA-FA on human-derived cancer cells was also examined. Two cancer cell lines were used in these studies: glioblastoma multiforme A172 and colon adenocarcinoma SW620 cell lines. A172 cells are characterized by high expression of proton-coupled folate transporter (PCFT),^23^ and they also express CD44 receptor.^15^ SW620 cells also express folate receptor,^54^ but its expression is considerably lower. It was also shown that only about 6.1% of these cells are CD44 positive.^55^ The results of the MTT assay are presented in Figure 11. The analysis revealed that the response of the cells differs visibly, based on the cell line studied and the SPIONs’ coating. For the A172 cell line after 24 h at the highest Fe concentration tested (100 μg/mL), SPION/HA (Figure 11A) decreased the viability of the cells to (81 ± 5) %, while for SPION/HA-FA, the viability was much lower, namely (59 ± 3) % (Figure 11B). The internalization of the nanoparticles is increased due to the presence of both CD44 and PCFT on the surface of the A172 cells,^23^ resulting in the greater biological response^56^ in comparison to nonfunctionalized SPION/HA. Relatively high cell viability, around 97%, was observed for SPION/HA for the Fe concentration up to 50 μg/mL. For FA-coupled NPs, the viability decreased to (90 ± 1) % already in the lowest Fe concentration tested (1 μg/mL). After 48 and 72 h, further viability drops are observed for both types of tested SPIONs. In the case of SW620 cell line (Figure 11C,D), there is a similar trend, namely, SPION/HA-FA nanoparticles cause concentration-dependent decrease in the viability of cells already at 5 μg/mL, while for SPION/HA, a dose-dependent decrease was only registered at the concentrations of 25 μg/mL and higher. Somewhat surprisingly, SW620 cells were more sensitive to both SPION systems than A172 cells, even though the CD44 receptor is much less expressed in this cell line. Such a significant increase in the observed cell death corresponds well with recent reports on the ferroptotic effect of iron-based particles in cells.^57−60^

#### 3.7.2 Cellular Uptake–Confocal Microscopy

**Figure 12:**
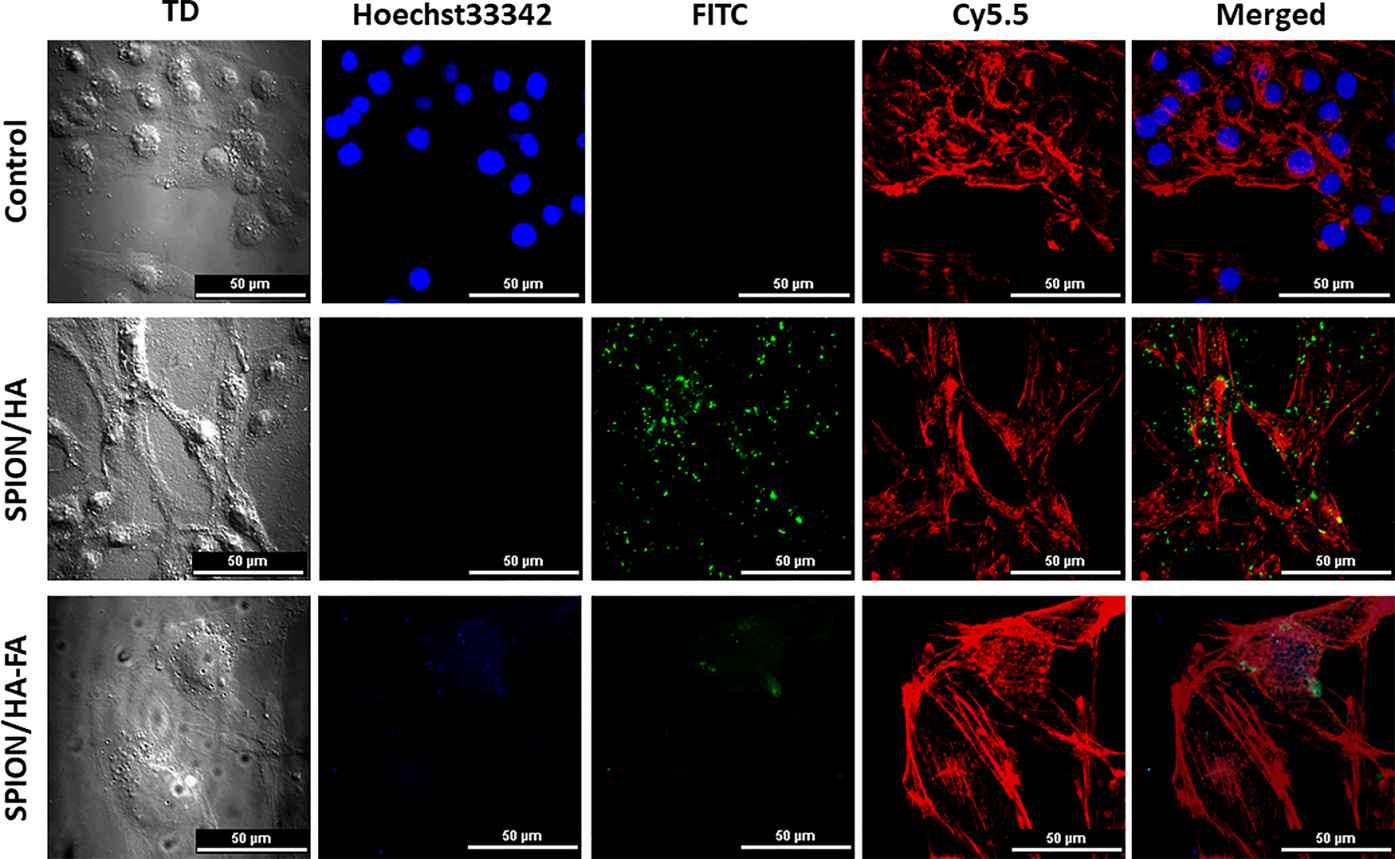
Confocal laser scanning microscope images of A172 cells, incubated with FITC-labeled SPION/HA or SPION/HA-FA. Control: fluorescence image (40× objective) of the control cells with stained cell nuclei (blue fluorescence) and F-actin (red fluorescence); SPION/HA: fluorescence image (40× objective) of the cells with stained F-actin (red fluorescence) after incubation with FITC-labeled SPION/HA (green fluorescence); SPION/HA-FA: fluorescence image (100× objective) of the cells with stained F-actin (red fluorescence) after incubation with FITC-labeled SPION/HA-FA (blue and green fluorescence).

Human glioblastoma A172 cells were incubated with FITC-labeled SPION/HA and SPION/HA-FA and studied using confocal microscopy in order to assess the cellular uptake of nanoparticles. The obtained images are presented in Figure 12.

In the control cells, both actin filaments and nuclei were stained, while in the case of cells incubated with SPIONs, only actin was labeled. That is because the blue dye used to stain nuclei interfered with the fluorescence of the taken-up nanoparticles. After 6 h incubation of A172 cells with SPION/HA or SPION/HA-FA, the green fluorescence, originating from FITC attached to the nanoparticles’ surface, was observed entirely inside the cells, within cytoskeleton (F-actin filaments). That confirmed an effective uptake of both types of nanoparticles by the tested glioblastoma cells. For the FA-modified particles this fluorescence was less intense as less surface functional groups was available for the FITC labeling due to the FA modification. However, for SPION/HA-FA (but not SPION/HA), a blue fluorescence originating from FA^61^ was additionally detected, providing an additional proof of its successful attachment to SPION’s surface.

## 4 Conclusions

We developed a relatively simple procedure that allowed us to synthesize SPION/HA nanoparticles under conditions less stringent than those previously proposed in the literature. The average hydrodynamic diameter of our SPION/HA nanoparticles was about 137 nm, and the magnetic core size was about 7 nm. The measured zeta potential was −53 mV, providing excellent colloidal stability for our nanoparticles.

The magnetic cores of our SPIONs are composed of magnetite in its oxidized form, maghemite, and their Mössbauer spectrum showed a single broad line characteristic of relaxational spectra. SPION/HA were characterized by relatively low magnetocrystalline anisotropy. Based on magnetometry results, the superparamagnetic blocking temperature for SPION/HA was estimated at 100 K, and magnetic saturation was achieved at a magnetic field strength of 20 kOe. The stability in physiological media showed that the surface attachment of FA considerably improved the colloidal stability of the nanoparticles. SPION/HA-FA remained stable for 50 h at pH = 7.4.

In summary, the particles obtained were spherical in shape and had low dispersity, adequate size, and high colloidal stability. Both SPION/HA and SPION/HA-FA were superparamagnetic and could serve as effective T2 contrasts for MRI, although modification with folic acid significantly increased the proton relaxation rate (1/T2) in their water suspension. Indeed, the (1/T2) values for SPION/HA-FA exceeded those obtained for the commercial contrasts tested (Ocean and FeREX), and for the bimodal SPION-Gd contrasts described in the literature. The MRI scan carried out on phantoms containing various concentrations of SPION/HA and SPION/HA-FA further confirmed their high effectiveness as the MRI contrast agents. Biological studies showed that both SPION/HA and SPION/HA-FA are cytocompatible. However, a mild toxic effect toward hepatocytes was also observed for FA-functionalized nanoparticles; thus, lower concentrations of these particles, below 25 μg/mL, should be used. Studies conducted on the A172 glioma cell line overexpressing the HA and folate receptor showed that both types of nanoparticles were efficiently taken up by these tumor cells. Cytotoxicity studies also showed that SPION/HA-FA could reduce tumor cell viability in a dose-dependent manner. Similar but more pronounced effects were observed with SW620 colon cells, possibly due to the higher sensitivity of this metastatic cell line to ferroptosis. Such an observation leads to the hypothesis that our FA-functionalized SPIONs may also provide therapeutic benefits. The resulting magnetic nanoparticulate systems, particularly the SPION/HA-FA nanoparticles, show significant potential as T2 MRI contrast agents for use in both tumor imaging and theranostics.

## Supporting Information Available

Additional physicochemical characteristics of obtained products: AFM measurements of the nanoparticles performed in water; NMR spectrum of hyaluronic acid; cryo-TEM image of SPION/HA-FA, UV–Vis spectra of: SPION/HA, SPION/HA-CYS, and SPION/HA-FA; coercivity; saturation magnetization and remanence values determined from magnetization vs magnetic field curves taken at a range of temperatures from 2 to 300 K; temperature dependence of the coercive field for the SPION/HA and magnetic remanence of the SPION/HA; and Mossbauer spectrum of the SPION/HA sample with a fit (PDF)
