# Supplementary material for: Hyaluronic Acid-Coated SPIONs with Attached Folic Acid as Potential T2 MRI Contrasts for Anticancer Therapies
Source: ACS Appl Mater Interfaces. 2025 Jan 29;17(6):9059–73. doi: 10.1021/acsami.4c20101 (PMC11826879; doi:10.1021/acsami.4c20101)
Supplement: Supplementary file 1 — am4c20101_si_001.pdf [file am4c20101_si_001.pdf]

Supporting Information

Hyaluronic acid-coated SPIONs with attached folic acid as potential T2 MRI contrasts for anticancer therapies

Martyna Kasprzyk[1,2], Gabriela Opiła[3], Alicja Hinz[4], Sylwia Stankiewicz [2,4], Monika Bzowska[4], Karol Wolski[1], Joanna Dulińska-Litewka[5], Janusz Przewoźnik[3], Czesław Kapusta[3], Anna Karewicz[1,*]

[1] Department of Chemistry, Jagiellonian University, Gronostajowa 2, 30-387 Kraków, Poland.

[2] Doctoral School of Exact and Natural Sciences, Jagiellonian University, Prof. S. Łojasiewicza 11, 30-348 Kraków, Poland.

[3] AGH University of Kraków, Faculty of Physics and Applied Computer Science, Al. A. Mickiewicza 30, 30-059 Kraków, Poland.

[4] Department of Cell Biochemistry, Faculty of Biochemistry, Biophysics and Biotechnology, Jagiellonian University, Gronostajowa 7, 30-387 Kraków, Poland.

[5] Chair of Medical Biochemistry, Jagiellonian University Medical College, Kopernika 7, 31-034 Kraków, Poland.

* Corresponding Author:

Anna Karewicz

Department of Chemistry, Jagiellonian University, Gronostajowa 2, 30-387 Kraków, Poland.

E-mail: karewicz@chemia.uj.edu.pl

1. AFM measurements of SPION/HA performed in water

Behaviour of the SPION/HA in water was also explored, revealing significant swelling of the NPs' coating. Different loading forces were applied for imaging. It could be seen, that low forces do not deform the particles and the structures appear to be larger than after imaging using greater forces (1.5 nN and 3.0 nN). Along with the increased loading forces, sample is deformed to the greater extent and particles' cores are imaged rather than swelled hyaluronic acid coating.

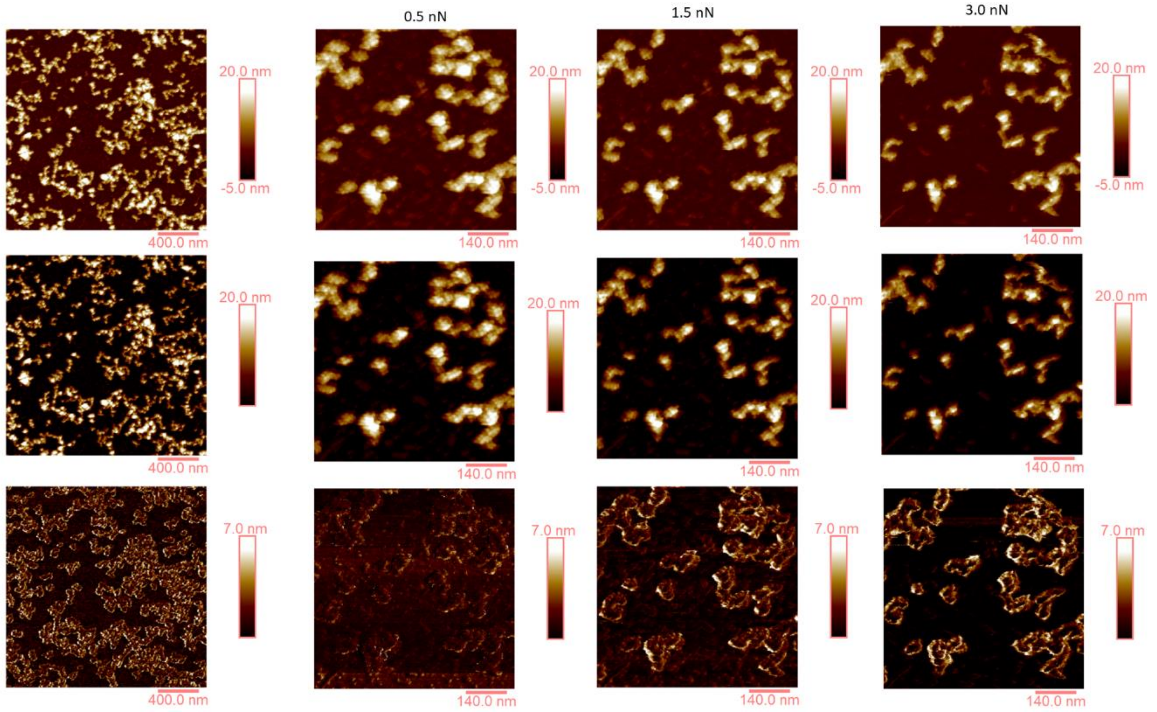

Figure S1 AFM images of SPION/HA. Measurements were performed in water. Varying loading forces were applied: 0.5 nN, 1.5 nN and 3.0 nN, respectively.

2. sHA NMR

Hyaluronic acid was characterised physiochemically after the sonication process, to asses if such preparation method influence the HA's structure. Thus, ^{1}H NMR spectrum of sHA was obtained. As could be seen, signal from hemiacetal proton-formed aldehyde is not present within 5.0-5.1 ppm region[1], confirming lack of the oxidation of the hydroxyl groups in glucuronic acid moiety.

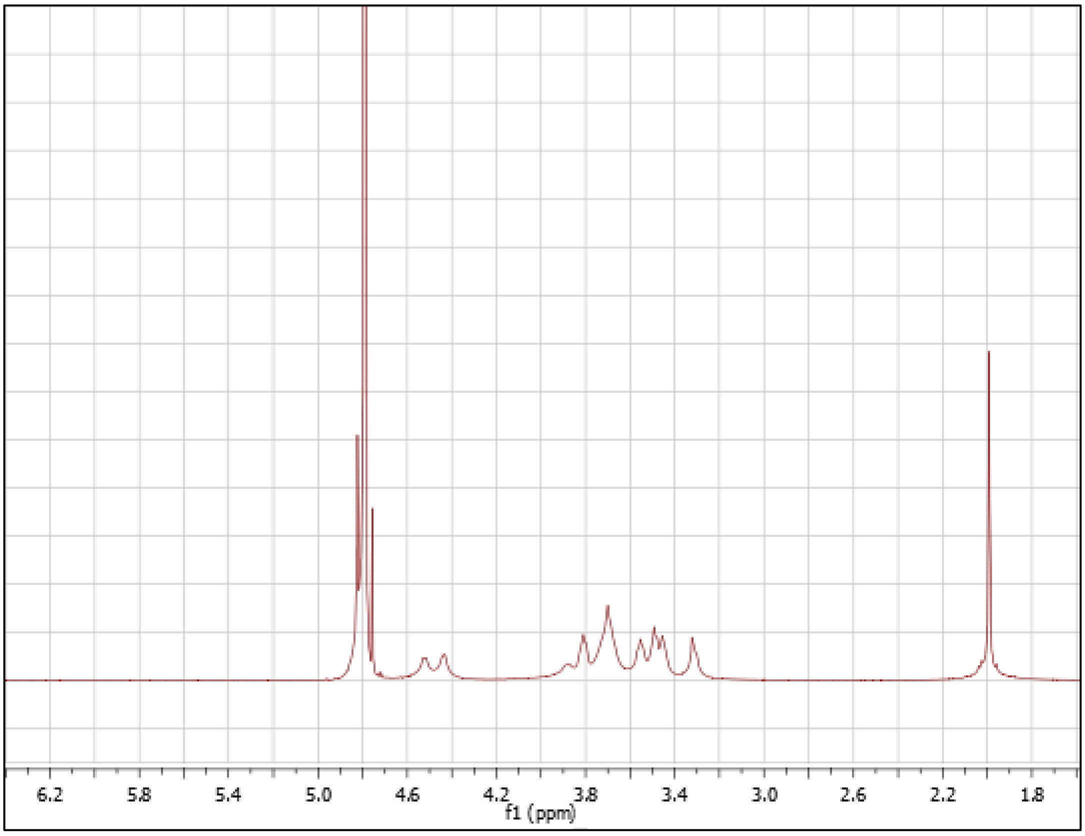

Figure S2 ^{1}H-NMR spectrum of the sHA in D_2O.

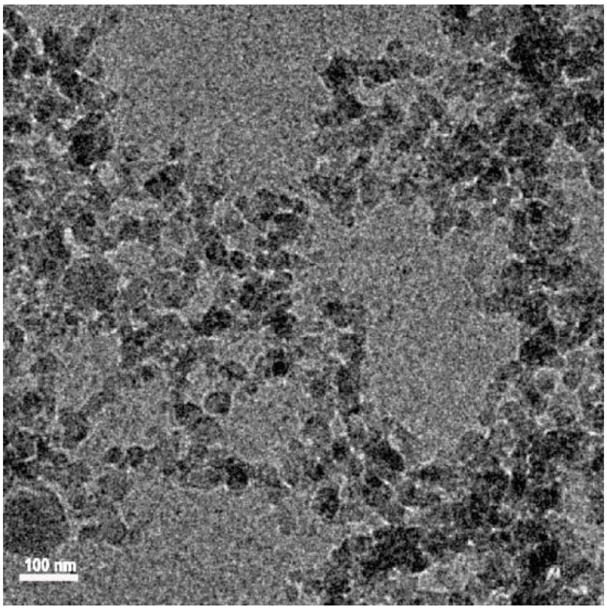

Figure S3 Cryo-TEM image of the SPION/HA-FA nanoparticles

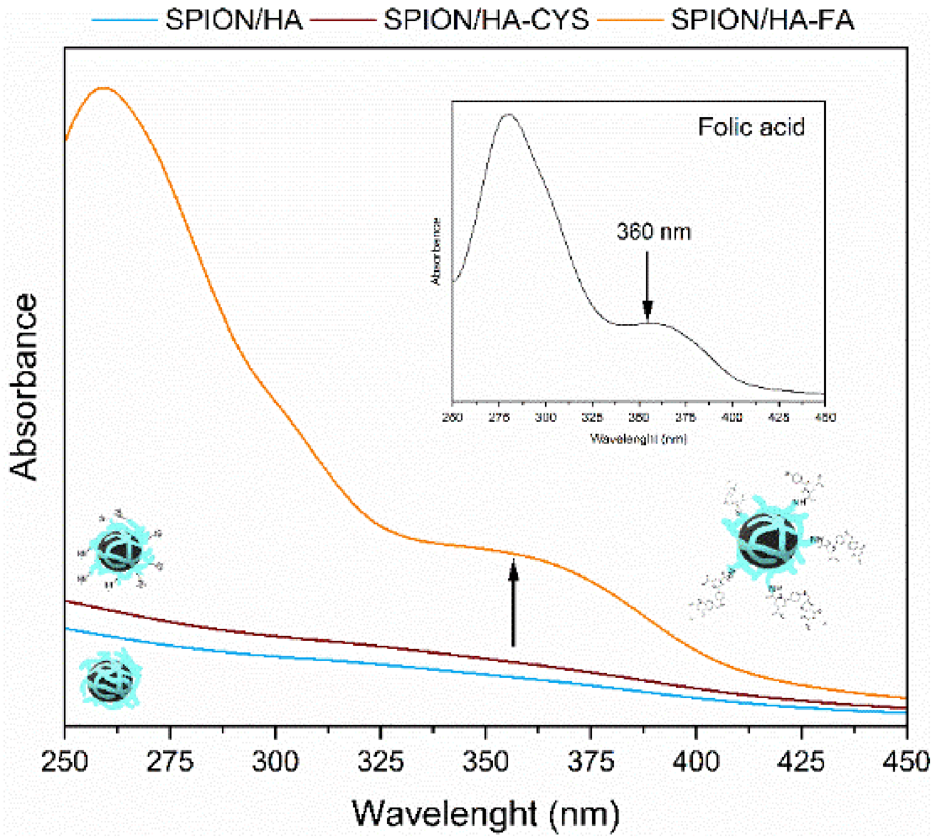

Figure S4. UV-VIS spectra of: SPION/HA, SPION/HA-CYS and SPION/HA-FA with the insert of UV-VIS spectrum of folic acid solution in water.

Table S1. Coercivity, saturation magnetization and remanence values determined from magnetization vs. magnetic field curves taken at a range of temperatures from 2 K to 300 K. The two coercivity values (-) and (+) are measured at the hysteresis loop intersection with a magnetic field axis at the negative and positive sides, respectively.

Temperature (K)	Coercivity (kOe) (-)	Coeercivity (kOe) (+)	Remanence (emu/g)	Saturation magnetisation (emu/g)
2	-0.18	0.18	15.28	69.38
4	-0.15	0.15	13.78	69.33
10	-0.10	0.10	8.90	69.20
25	-0.04	0.04	4.93	69.01
50	-0.01	0.01	1.64	68.63
100	0.00	0.00	0.00	67.36
150	0.00	0.00	0.00	65.58
200	0.00	0.00	0.00	63.24
300	0.00	0.00	0.00	57.30

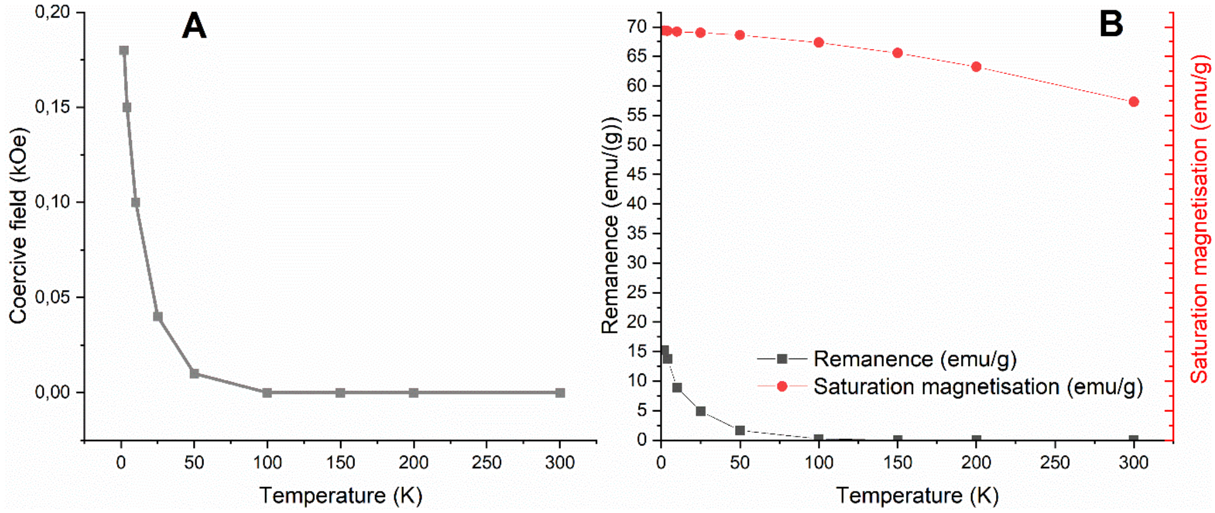

Figure S5. Temperature dependence of the values of the A) coercive field for the SPION/HA and B) magnetic remanence and saturation magnetization of the SPION/HA.

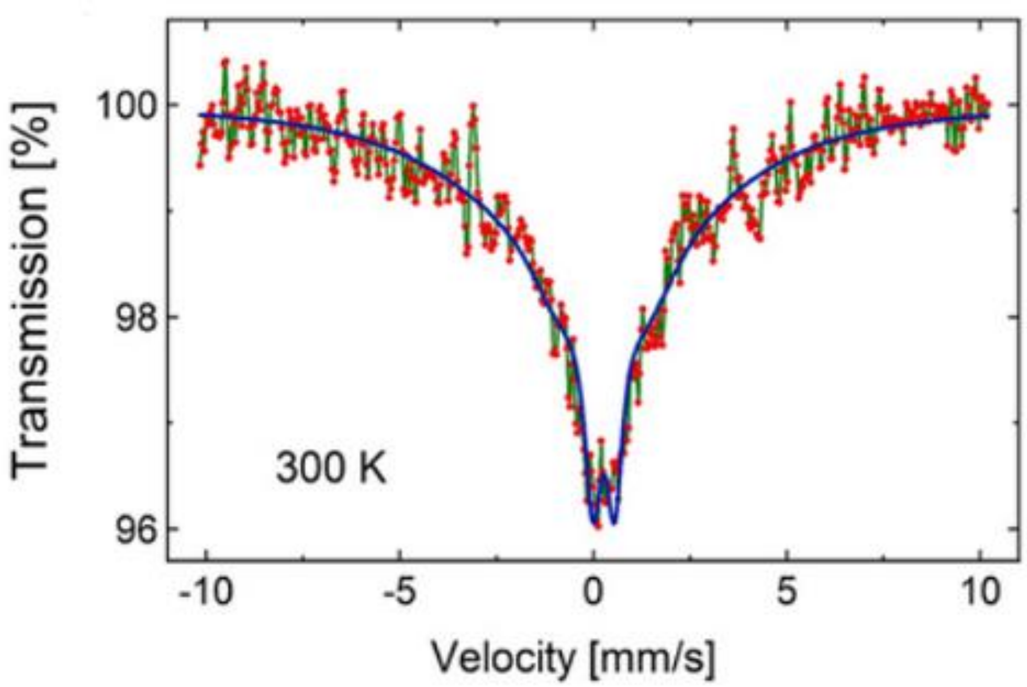

Figure S6. Mossbauer spectrum of the SPION/HA sample with a fit (solid blue line)

Mössbauer spectroscopy study was performed for the SPION/HA. The Mössbauer spectrum obtained at room temperature is presented in Figure S6 and contains a heavily broadened line with the shape characteristic of the relaxational spectra. It was fitted according to the model proposed by Tjon and Blume[2] and the fit is also shown in Figure S6. It provided the values of the fluctuation frequency f and relative mean residing time ρ of the magnetic moments of the nanoparticles along the easy magnetization direction

(termed as asymmetry parameter), amounting to f = (123 ± 43) MHz and ρ =(0.22 ± 0.03). In this case the asymmetry is low, so the lines of the magnetic sextets nearly collapse. The value of f is higher than in the case of nanoparticles synthesized by Gumieniczek-Chłopek et al.[3] in a thermal decomposition process, namely 52 MHz. Relatively low asymmetry parameter indicates rather low magnetocrystalline anisotropy.

References

[1] Joo H, Park J, Sutthiwanjampa C, Kim H, Bae T, Kim W, et al. Surface coating with hyaluronic acid-gelatin-crosslinked hydrogel on gelatin-conjugated poly(Dimethylsiloxane) for implantable medical device-induced fibrosis. Pharmaceutics 2021;13:1–21. https://doi.org/10.3390/pharmaceutics13020269.

[2] Tjon, J. A.; Blume, M. Mössbauer Spectra in a Fluctuating Environment II. Randomly Varying Electric Field Gradients. *Physical Reviews* **1968**, *165*, 456–461. https://doi.org/10.1103/PhysRev.165.456

[3] Gumieniczek-Chłopek, E.; Odrobinska, J.; Straczek, T.; Radziszewska, A.; Zapotoczny, S.; Kapusta, C. Hydrophobically Coated Superparamagnetic Iron Oxides Nanoparticles Incorporated into Polymer-Based Nanocapsules Dispersed in Water. *Materials* **2020**, *13* (5). https://doi.org/10.3390/ma13051219
